# Bioinformatics analysis of whole slide images reveals significant neighborhood preferences of tumor cells in Hodgkin lymphoma

**DOI:** 10.1371/journal.pcbi.1007516

**Published:** 2020-01-21

**Authors:** Jennifer Hannig, Hendrik Schäfer, Jörg Ackermann, Marie Hebel, Tim Schäfer, Claudia Döring, Sylvia Hartmann, Martin-Leo Hansmann, Ina Koch

**Affiliations:** 1 KITE - Kompetenzzentrum für Informationstechnologie, Technische Hochschule Mittelhessen, Friedberg, Germany; 2 Molecular Bioinformatics, Institute of Computer Science, Johann Wolfgang Goethe-University, Frankfurt am Main, Germany; 3 Institute of Biochemistry II, Johann Wolfgang Goethe-University, University Hospital Frankfurt am Main, Frankfurt am Main, Germany; 4 Department of Child and Adolescent Psychiatry, University Hospital Frankfurt am Main, Johann Wolfgang Goethe-University, Frankfurt am Main, Germany; 5 Dr. Senckenberg Institute of Pathology, Johann Wolfgang Goethe-University, Frankfurt am Main, Germany; 6 Consultation and reference center for lymph node pathology at Dr. Senckenberg Institute of Pathology, Johann Wolfgang Goethe-University, Frankfurt am Main, Germany; University of Virginia, UNITED STATES

## Abstract

In pathology, tissue images are evaluated using a light microscope, relying on the expertise and experience of pathologists. There is a great need for computational methods to quantify and standardize histological observations. Computational quantification methods become more and more essential to evaluate tissue images. In particular, the distribution of tumor cells and their microenvironment are of special interest. Here, we systematically investigated tumor cell properties and their spatial neighborhood relations by a new application of statistical analysis to whole slide images of Hodgkin lymphoma, a tumor arising in lymph nodes, and inflammation of lymph nodes called lymphadenitis. We considered properties of more than 400, 000 immunohistochemically stained, CD30-positive cells in 35 whole slide images of tissue sections from subtypes of the classical Hodgkin lymphoma, nodular sclerosis and mixed cellularity, as well as from lymphadenitis. We found that cells of specific morphology exhibited significantly favored and unfavored spatial neighborhood relations of cells in dependence of their morphology. This information is important to evaluate differences between Hodgkin lymph nodes infiltrated by tumor cells (Hodgkin lymphoma) and inflamed lymph nodes, concerning the neighborhood relations of cells and the sizes of cells. The quantification of neighborhood relations revealed new insights of relations of CD30-positive cells in different diagnosis cases. The approach is general and can easily be applied to whole slide image analysis of other tumor types.

## Author summary

In pathology, histological diagnosis is still challenging, in particular, for tumor diseases. Pathologists diagnose the disease and its stage of development on the basis of evaluation and interpretation of images of tissue sections. The quantification of experimental data to support decisions of diagnosis and prognosis, applying bioinformatics methods, is an important issue. Here, we introduce a new, general approach to analyze tissue images of tumor and non-tumor patients and to evaluate the distribution of tumor cells in the tissue. Moreover, we consider neighborhood relations between immunostained cells of different cell morphology. We focus on a special type of lymph node tumor, the Hodgkin lymphoma, exploring the two main types of the classical Hodgkin lymphoma, the nodular sclerosis and the mixed cellularity, and the non-tumor case, the lymphadenitis, representing an inflammation of the lymph node. We considered more than 400, 000 cells immunohistochemically stained with CD30 in 35 whole slide images of tissue sections. We found that cells of specific morphology exhibited significant relations to cells of certain morphology as spatial nearest neighbor. We could show different neighborhood patterns of CD30-positive cells between tumor and non-tumor. The approach is general and can easily be applied to other tumor types.

## Introduction

The lymph node is a structured organ with major compartments, such as the subcapsular sinus, B cell follicles, the T cell zone, trabecular and medullary sinuses, and blood vessels. Many cells of different type enter the lymph node. They migrate from compartment to compartment, interact with each other and with other cells, and show a complex movement in a stromal cell network [1]. This movement is neither systematically investigated nor understood. In particular, the movement of tumor cells would be of great interest to comprehend the progress of the disease.

Hodgkin lymphoma (HL) is a malignant tumor disease of the lymphoid system, which originates from B-lineage cells at various stages of development [2]. The annual incidence of about three cases per 100, 000 persons makes HL to one of the most frequent lymphoma of the Western civilization [3, 4]. In the United States, about 185, 000 people were diagnosed with HL in 2011. Currently, more than 9, 000 new cases are estimated per year. The probability to be diagnosed with HL for a single person during the life time is 0.2%. 85.3% of the HL patients survive five years or more, which is a high survival rate compared to other tumor types. Nevertheless, 1, 100 people die of HL per year in the United States, so HL is still a severe disease.

About 11% of all malignant lymphomas are HL. The WHO (World Health Organization) classification differentiates HL into two main groups [5], classical Hodgkin lymphoma (cHL), the most common type with about 95% of all cases, and the less frequent nodular lymphocyte predominant type. Classical Hodgkin lymphoma is further subdivided into four types. The nodular sclerosis type (NScHL) is the most common type with 60 − 70% of all cHL cases. In NScHL, at least one nodule formed by sclerotic bands, which contains malignant tissue, is present. With roughly 25%, mixed cellularity cHL (MCcHL) is the second frequent type of cHL. The two other subtypes named lymphocyte-depleted and lymphocyte-rich cHL are rare. They will not be considered in this study.

**Fig 1:**
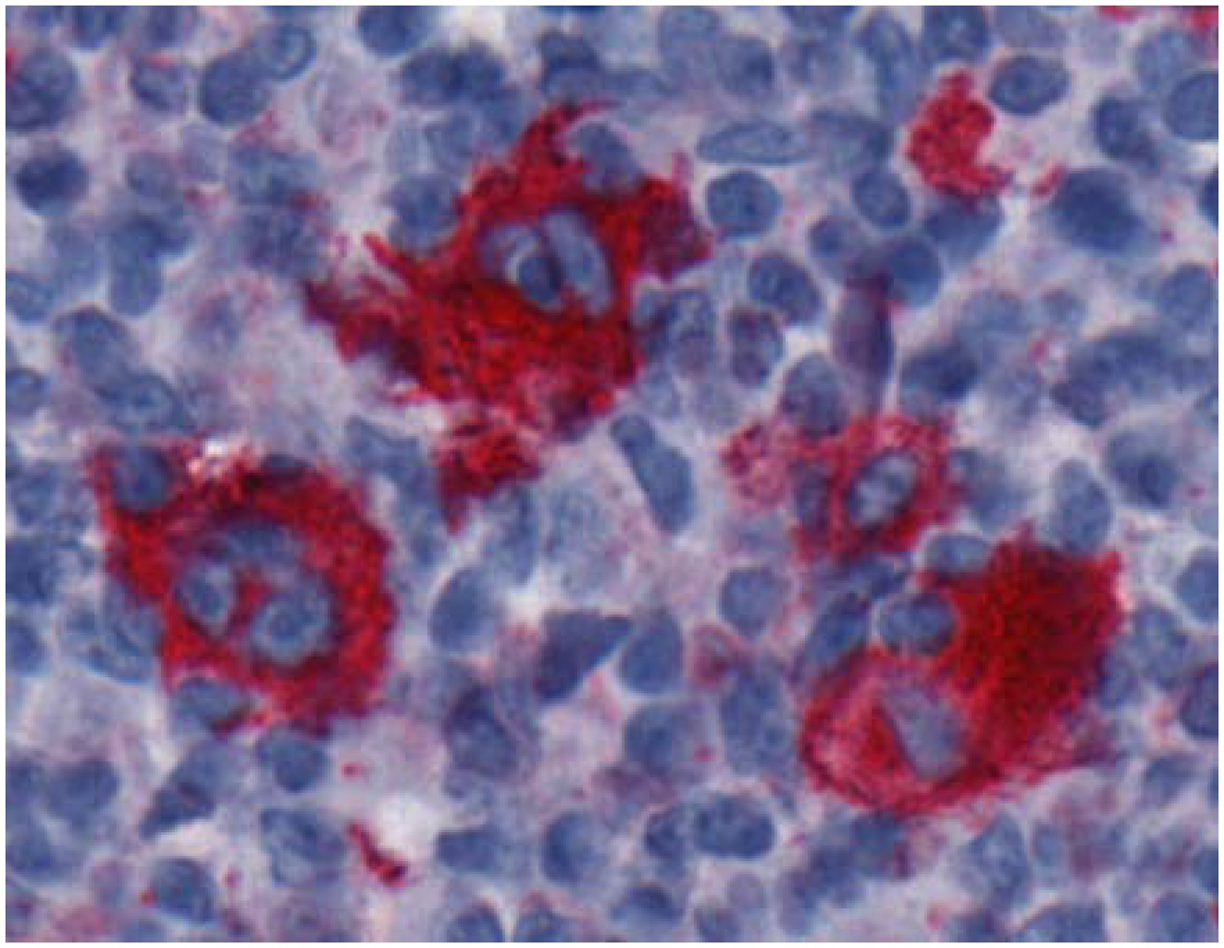
Reed-Sternberg cells. Cells with CD30 immunostaining are colored red. There is one trinucleated HRS cell in the left part and one binucleated HRS cell in the middle upper part. The cell nuclei colored blue are stained using hematoxylin.

On cellular level, HL has some unusual characteristics compared to other tumors. The malignant morphologically huge pleomorphic cells called Hodgkin and Reed-Sternberg (HRS) cells originate mainly from B cell pre-cursor cells of the germinal center. In rare cases, HRS cells originate from T cells. Whereas Hodgkin cells are mononucleated, Reed-Sternberg cells are multinucleated (Fig 1).

HRS cells exhibit a broad morphological spectrum. In contrast to other tumor types, for cHL, only 1 to 2% of the tissue of the lymph node is covered by tumor cells. The majority of the tumor microenvironment consists of reactive cells of the immune system, including lymphocytes, macrophages, eosinophiles, mast cells, plasma cells, and stromal cells. In consequence, no solid tumor is present, but an inflammatory heterogeneous microenvironment. HRS cells actively influence their environment via rearrangement of cytokines and chemokines [6]. Quantified knowledge of the distribution and movement of tumor cells is necessary to understand the progress of the disease.

For diagnosis, tissue sections are cut and immunohistochemically stained. The visualization of HRS cells by specific staining based on CD30 antibody in combination with microscopy is routinely applied for diagnosis of lymphoma, see subsection *Image processing* in section *Materials and Methods* for more detail. Computer-aided analysis of tissue sections becomes essential for diagnosis and prediction of the disease’s progress. All this led to the emerging field of digital pathology, see [7–9]. Nowadays, beside traditional microscopy, digital scanners allow the digitization of whole object slides to whole slide images (WSI). Nevertheless, WSI are rarely used in diagnostic work-flows, because it would require a costly infrastructure to automate the WSI retrieval process. Another aspect is that, the visual evaluation of a pathologist is strongly dependent from the display used. Although a few institutions have already implemented a digital workflow, see for example [10, 11], the majority of pathologists still evaluate tissue sections in the microscope.

For WSI analysis, Kothari *et al*. applied a quantitative analysis of WSI to explore kidney renal clear cell carcinoma cases [12]. In previous work, we have investigated the quantification of CD30-positive pixels in cHL WSI [13]. These tissue sections have shown a high variability regarding the CD30-positive pixel count, which most likely has been an effect of the various progression states of the disease. Therefore, a classification based on pixel quantification has not been able to distinguish between the disease types, MCcHL and NScHL, and the non-tumor type, lymphadenitis.

Malignant cells have been reported to develop abnormal, irregularly shaped nuclei [14, 15]. To separate and label benign and malignant tissues, morphologic features, such as gray-level texture features, color-based features, Law’s Texture Energy-based features, Tamura’s features, and wavelet features, have been applied [16–20]. These texture-based methods consider the neighborhood of cells, applying the gray scale co-occurrence matrix, the wavelet transformation, and Fourier transformation, see [21] for a detailed overview. Various imaging approaches have been applied to analyze and classify malignant tissues for Barrett’s cancer and prostate cancer, respectively [19, 22]. These previous approaches have analyzed color, texture, and object features, but no morphological shape features of tumor cells. Image texture analysis has been also applied to breast tissue images to determine the region of interest to follow up microarray experiments [23, 24]. Other work concerns heterogeneity assessment of tissue sections in WSI to label breast cancer histopathological digital images [25].

Currently, more and more deep learning methods are applied in digital pathology, e.g. convolutional neural networks, see, for example, [26–28] for more information and use cases. Deep learning methods provide a very effective and versatile way for the analysis and classification of tumor images. The drawbacks of these methods are the necessity of large training sets and the loss of traceability and, thus, the lack of full interpretability of the results.

In the diagnostic processes, a systematic computational analysis is not yet regularly applied [29]. There are no statistical studies available that investigate morphological properties and neighborhood relations of tumor cells in the lymph node. The aim of the present study was to draw conclusions about migration of HRS cells by systematically analyzing the distribution of HRS cells in the tissue as a function of their size and shape. This includes also non-tumor cells, which are CD30-positive and represent activated lymphocytes. An inflammation of the lymph node called lymphadenitis (LA) often contains such activated lymphocytes. It is so far not possible to perform life imaging of human lymph nodes affected by HL, and a corresponding mouse model of HL does not exist. Since histological sections represent snapshots of the tumor development, including migration, we explored features in the WSI, such as the number, distance, and neighborhood relationship of tumor cells, applying statistical methods.

We wanted to answer following questions. Do HRS cells come in close spatial contact to communicate and cooperate with each other? Is the close spatial neighborhood somehow related to the morphology of the cells? Can we draw conclusions regarding the movement of HRS cells? We applied following hypotheses.

- To yield statistically significant neighborhood relations, we can apply a classification of cells to one of eight classes according to empirically defined specific thresholds for the morphological features eccentricity, solidity, and size.
- The morphology of the next neighbor of a CD30-positive cell depends statistically significant on the morphology of the cell itself.
- The cell morphology of a tumor cell is important to understand its role in the spatial organization and the spread of classical Hodgkin lymphoma.
- The size of CD30-positive cells is larger in classical Hodgkin lymphoma than in lymphadenitis.
- The majority of HRS cells in classical Hodgkin lymphoma have large diameters, i.e., diameters larger than 30 *μ*m.
- HRS cells of elongated shape are moving.
- Frayed cells are more communicating than non-frayed cells.
- Next neighbor relations of CD30-positive cells in tissue of patients with lymphadenitis and classical Hodgkin lymphoma are not random.
- CD30-positive cells cluster in classical Hodgkin lymphoma and lymphadenitis.
- The neighborhood relations of CD30-positive cells show differences between lymphadenitis and the subtypes nodular sclerosis and mixed cellularity of classical Hodgkin lymphoma.

To the best of our knowledge, these questions have not been addressed so far, neither for HL nor for other tumor types.

## Results and discussion

Here, we use abbreviations we describe in more detail in section *Materials and Methods*. We define a *profile class (PC)* for each cell, according to the size and morphology of the cell. We call the PC of the nearest neighbor of a cell as *neighbor profile class (NPC)*.

### Small cells occur much more frequent than large cells

**Fig 2:**
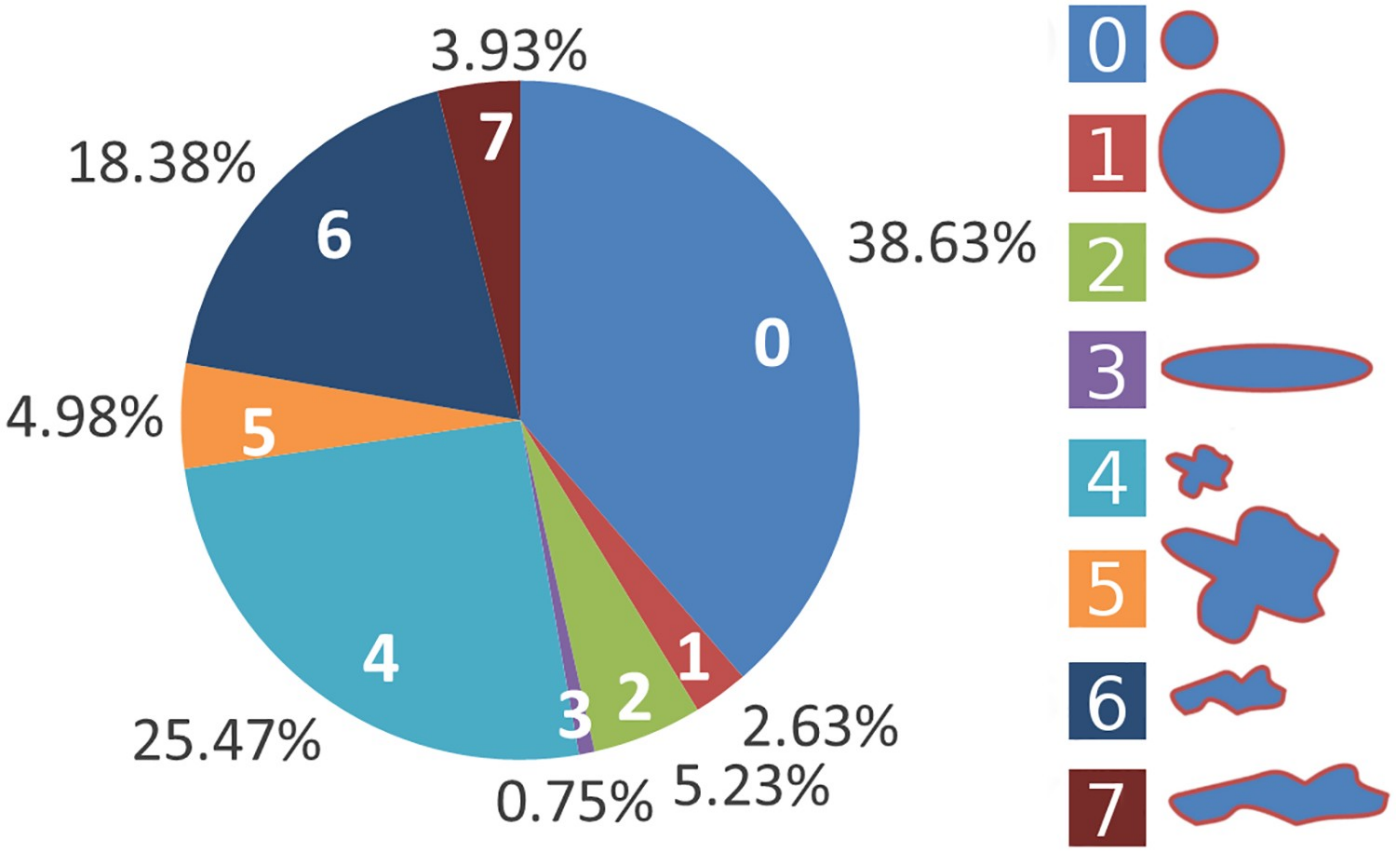
Fractions of cells. The fractions of cells classified according to the *PC* = 0 to *PC* = 7 averaged over the 35 images.

Fig 2 shows the fraction of PC averaged over the 35 images. *PC* = 0 is the most frequently occurring PC with 38.63%, describing small, round cells followed by *PC* = 4 with 25.47%, standing for also small, but frayed cells. Only very few cells, 0.75%, were large and elongated (*PC* = 3). Overall, *PC* = 1, 3, 5, and 7, describing large cells, were less frequent than the *PC* = 0, 2, 4, and 6, describing small cells. For disease-specific numbers for the fractions of PC, see S1 Table.

**Fig 3:**
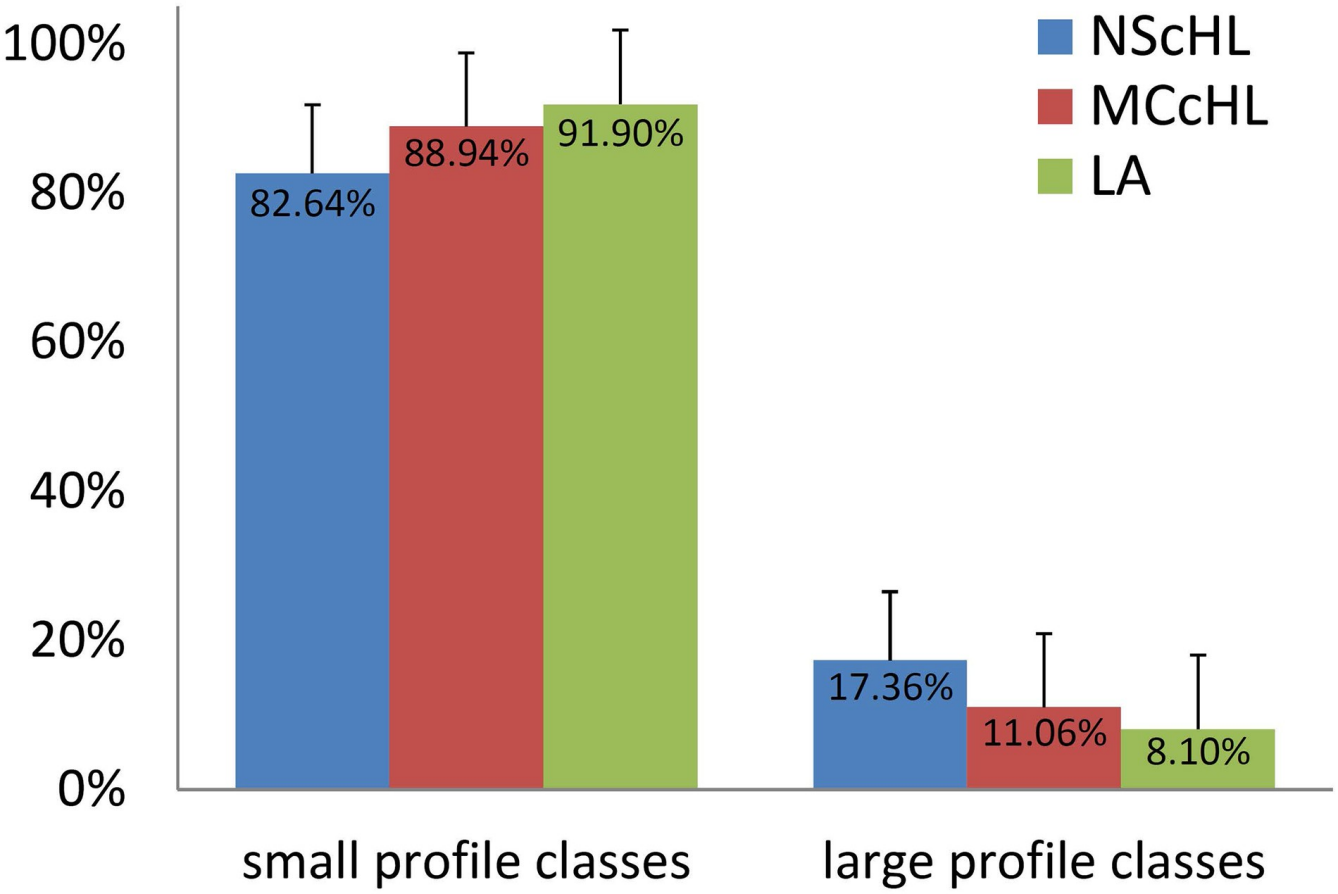
The fractions of small and large cells. The fractions of small (*PC* = 0, 2, 4, 6) and large (*PC* = 1, 3, 5, 7) PC as function of the diagnosis. The error bars show the standard deviation. The fraction of large PC in images of NScHL, MCcHL, and LA were statistically indistinguishable.

For NScHL and MCcHL, the fractions of large cells were 17.36% and 11.06%, respectively, whereas for LA only 8.10%, see Fig 3. The small cell profiles built the majority of the cells with 82.64% in NScHL, 88.94% in MCcHL, and 91.90% in LA.

Because cHL is commonly characterized by the occurrence of large HRS cells, we expected to find many large cells, at least in images diagnosed as cHL. Surprisingly, the measured difference in the numbers of large PC in cHL and LA were not sufficiently significant to distinguish between cHL and inflammation.

### The diameters of cells differ in classical Hodgkin lymphoma and lymphadenitis

**Fig 4:**
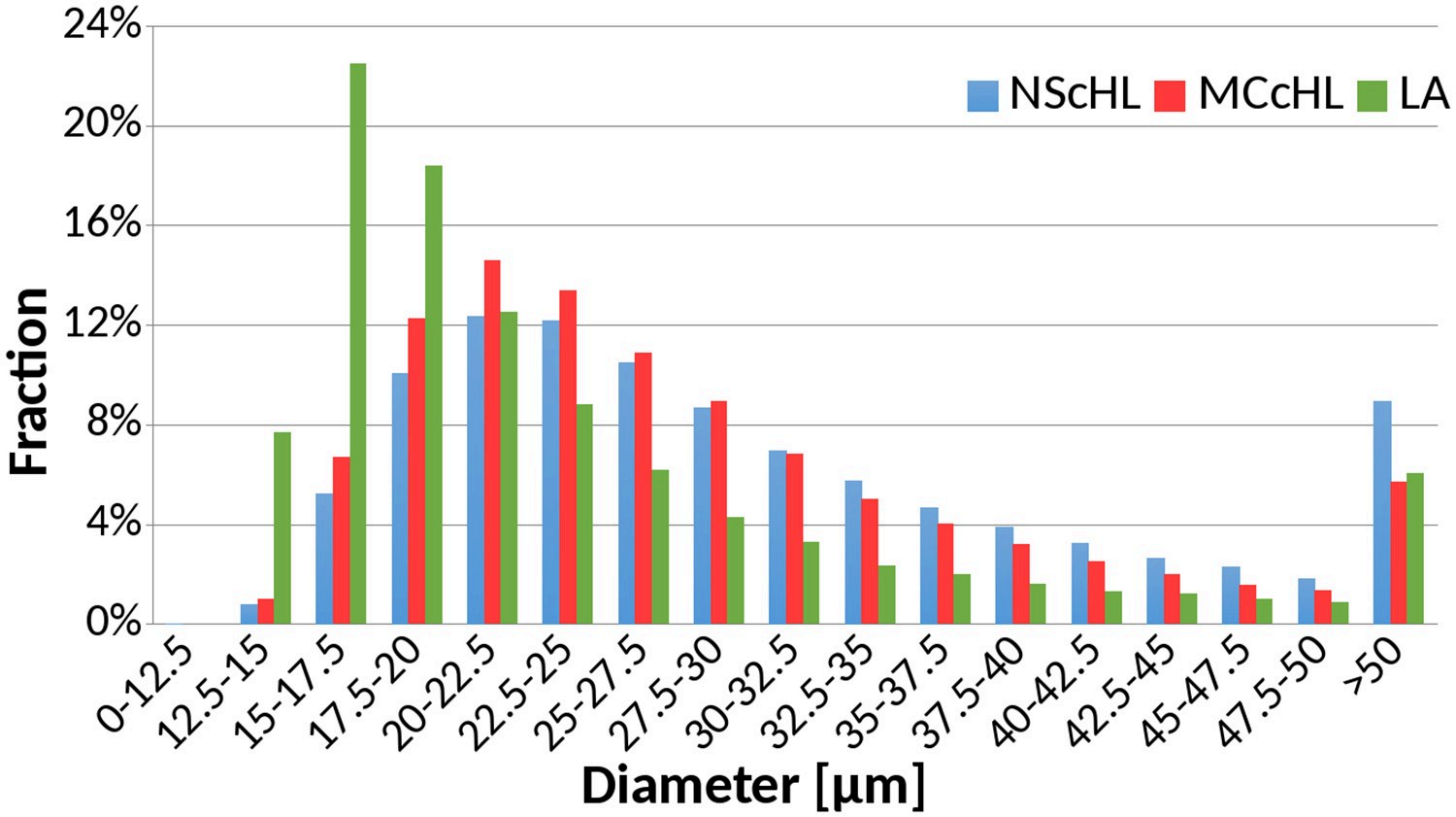
The diameter distribution of cells. The bar chart shows the distribution of the diameters for all diagnoses.

CD30-positive cells of the classical HL, NScHL and MCcHL, which are most likely HRS cells, vary in size between 20 and 60 *μm* [30–32], whereas the size of CD30-positive cells of LA, which originate from activated lymphocytes, was between 10 and 30 *μm* [33–35]. Based on the distribution of the cell diameters of CD30–positive cells (Fig 4), we assumed the majority of cells with a diameter in the range of 12.5 to 15 *μm* to originate from activated lymphocytes. Based on the ratio of the distributions at 12.5 to 15 *μm*, we estimated the proportion of HRS cells to be 89.8% and 87.0% for NScHL and MCcHL, respectively.

**Fig 5:**
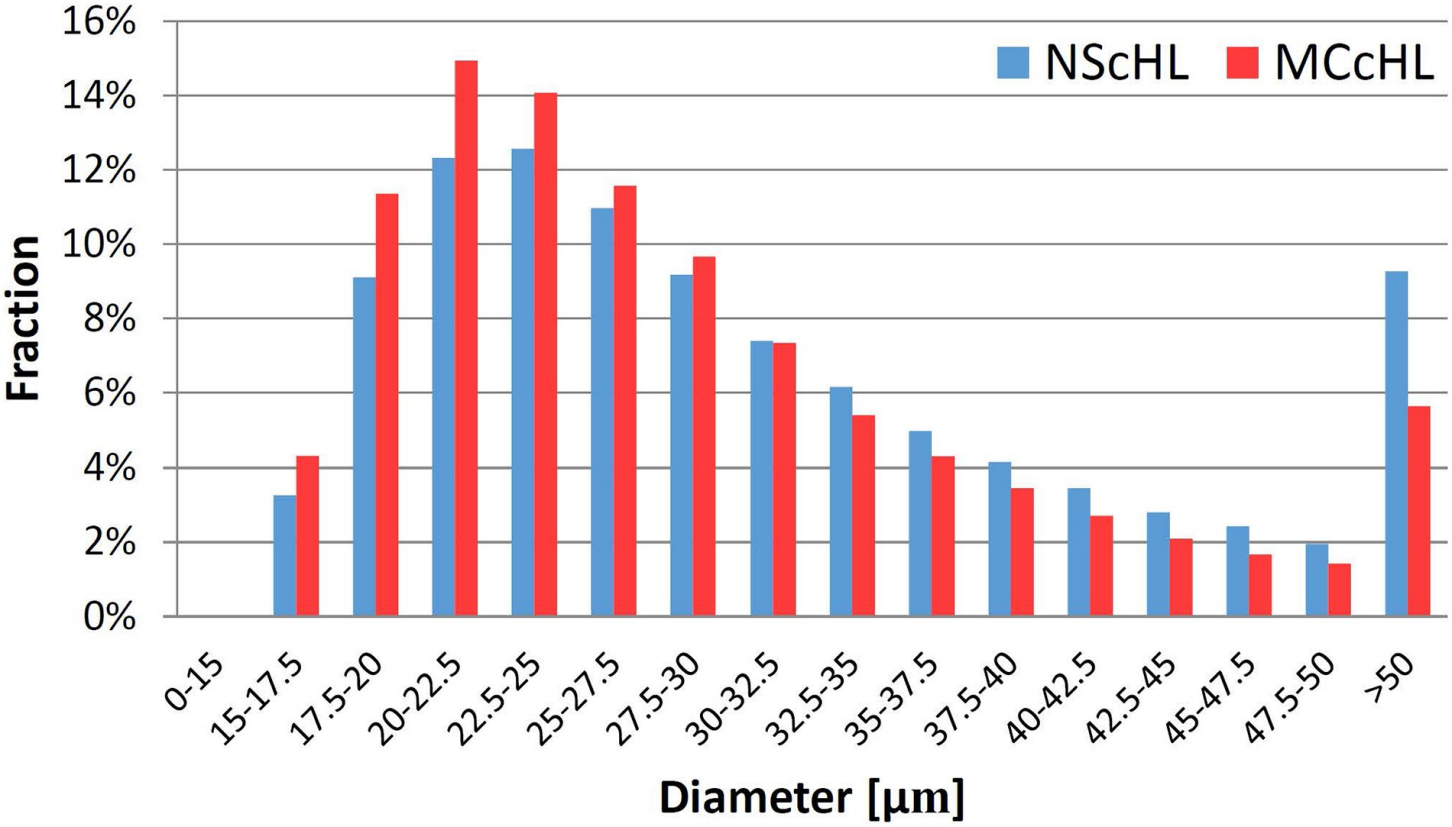
The corrected diameter distribution of cells. The bars show the relative fractions of HRS cells for the diagnoses NScHL and MCcHL, respectively. The relative fractions were corrected for the background of activated lymphocytes, see text.

Note that, not all CD30-positive cells are tumor cells, but might be activated lymphocytes and other kinds of cells. A pathologist would not classify these cells as HRS cells. To make the results comparable with the literature values in [30–32] obtained by visual inspection of high resolution electron microscopy images, we used the diameter distribution of LA and subtract the background of smaller cell profiles for NScHL and MCcHL, see Fig 5. These corrected distributions for NScHL and MCcHL have both a maximum for diameters in the range of 22.5 and 25 *μm*. For NScHL, the mean cell diameter was 30.6 *μm* with a large standard deviation of 10.2 *μm*, whereas the mean value for MCcHL was slightly smaller, 28.6 *μm* with a standard deviation of 9.3 *μm*. A large fraction of 9.2% and 5.6% of cells in NScHL and MCcHL, respectively, had a cell diameter greater than 50 *μm*.

The statistical analysis of a high number of CD30-positive cells offers a complementary view to a high-quality, visual definition of CD30-positive cells provided in the literature. Despite the different approaches, the values for the diameters of HRS cells, e.g. see Fig 5, and the fraction of large HRS cells with diameters greater than 50 *μm* were in perfect accordance with the literature [30–32].

### Small, round cells favor cells of the same type as neighbors

**Fig 6:**
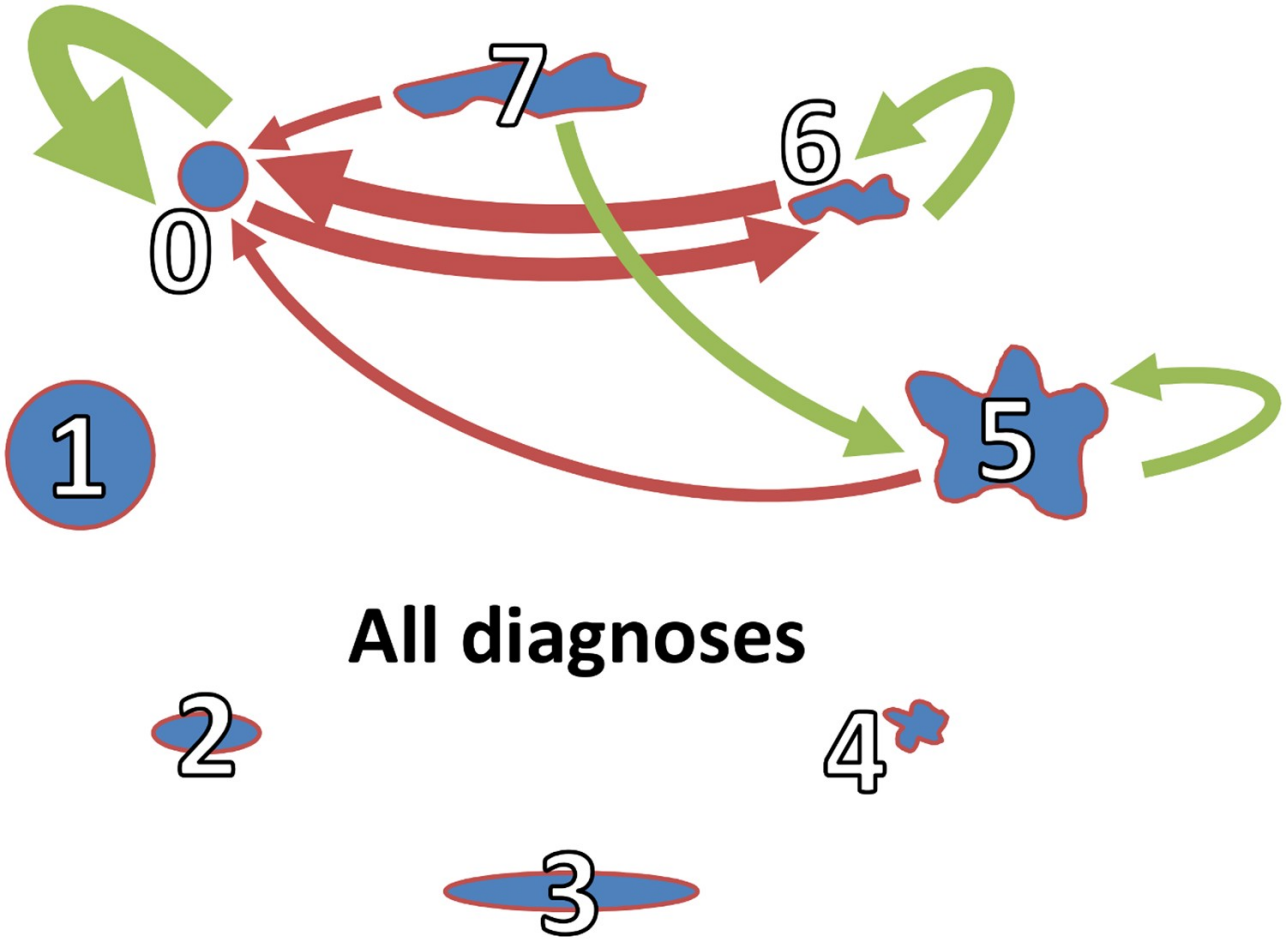
Neighborhood relations for all diagnoses. Each node represents one of the eight PC. Arrows are drawn between nodes if the absolute value of the score of neighborhood relation exceeds 50% of the maximally possible value. Green and red arrows represent favored and unfavored neighborhood relations, respectively. The thickness of the arrows correlates with the absolute value of the score. Thick green and red arrows stand for large positive and large negative scores, respectively.

In Fig 6, the thick red arrow from *PC* = 6 to *NPC* = 0 indicates a strong unfavored relation of small, elongated, frayed profiles with small, round cells in 74% of the images. In 26 of the 35 images, small, round cells were significantly underrepresented in the neighborhood of small, elongated, frayed PC. This was mutually, indicated by a reversed red arrow (from *PC* = 0 to *NPC* = 6) with a negative score of 69%. Note that, favored and unfavored neighborhood relations have not to be mutual, see S1 Fig.

In Fig 6, the green arrow from *PC* = 7 to *NPC* = 5 expresses the preference of large, elongated, frayed cells to have large, frayed cells in the neighborhood indicated by a positive score of 57%. Green loop arrows show the preferences of *PC* = 0 (small, round), *PC* = 6 (small, elongated, frayed), and *PC* = 5 (large, frayed) for cells of the same type as neighbors. In 89% of the images, small, round cells (*PC* = 0) favored cells of the same class as neighbors, *NPC* = 0, expressed by a significantly high number of this combination of the two classes in 31 images, see S2A) Fig. PC that show unfavored neighborhood relations to other classes (*PC* = 0, *PC* = 5, *PC* = 6) exhibit a favored neighborhood relation to cells of the same type.

### Neighborhood relations differ for the three diagnoses

**Fig 7:**
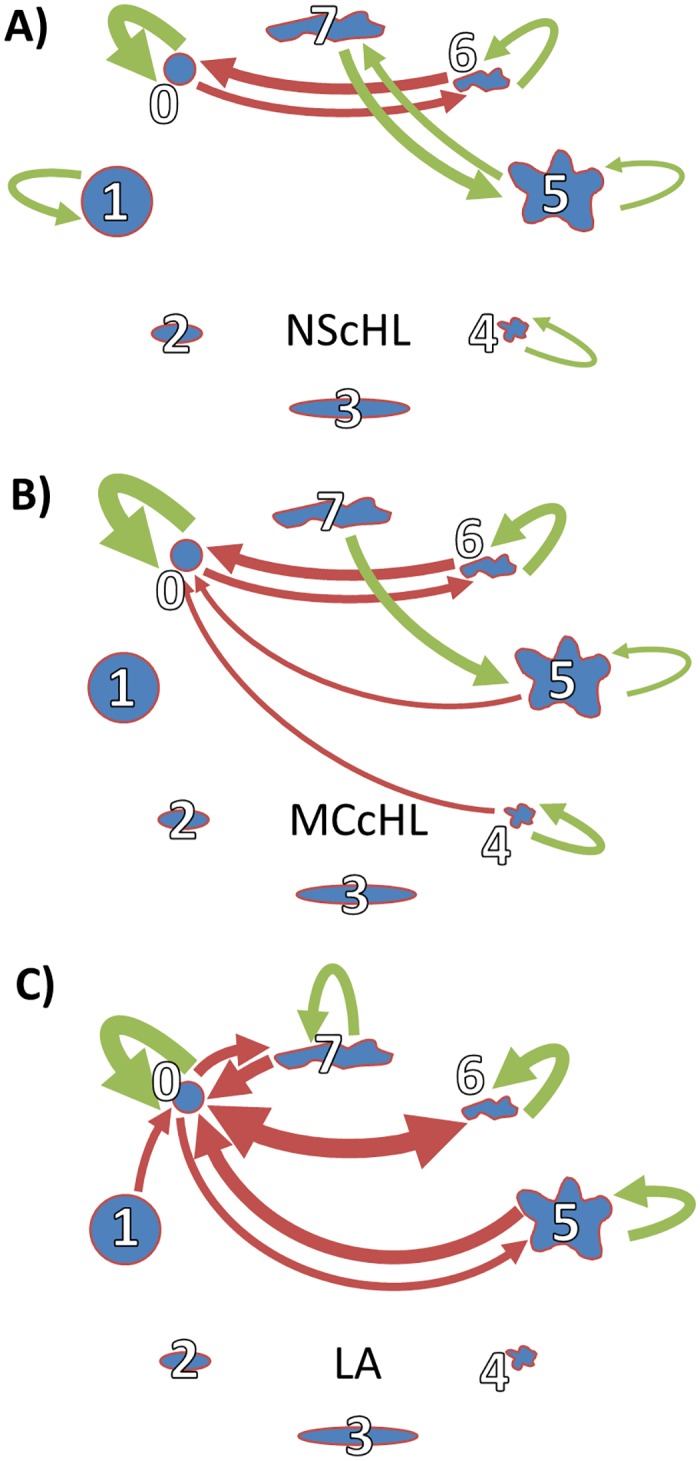
Disease-specific neighborhood relations. Networks of neighborhood relations A) in NScHL, B) in MCcHL and C) in LA. Each node represents one of the eight PC. The thickness of the arrows correlates with the absolute value of the score. Green and red arrows represent favored and unfavored neighborhood relations, respectively.

The three networks in Fig 7 illustrate the favored and unfavored neighborhoods of cells for the three diagnoses NScHL, MCcHL, and LA. The thickness of the arrows is proportional to the score, see S2 Fig. In all diagnoses, the small, round cells prefer cells of the same type as neighbors, exhibiting high positive scores of 83 to 92%, see S2 Fig. The neighborhood of small, round cells to other PC is always unfavored, in particular by small, elongated, frayed cells (*PC* = 6). No PC was observed to favor the small, round cells. Despite of common characteristics of the networks in Fig 7, significant differences in the neighborhood relation were visible for all diagnoses.

*NScHL*: Unfavored neighborhood relations of cells to other PC is less noticeable, see Fig 7A). Only two red arrows indicate a negative score of more than 50%. Small, round cells (*PC* = 0) show unfavored neighborhood relations to small, frayed, elongated cells (*PC* = 6) and *vice versa*. There exists a preferred neighborhood relation between large, frayed, elongated cells (*PC* = 7) and large, frayed cells (*PC* = 5).
*MCcHL*: As for NScHL, cells of *PC* = 7 (large, elongated, frayed) favor cells of *PC* = 5 (large, frayed), see Fig 7B). In contrast to MCcHL, small, round cells, *PC* = 0, were not preferred in their neighborhood.
*LA*: Fig 7C) shows many unfavored neighborhood relations between PC. For example, cells of *PC* = 0 (small, round) do not favor *PC* = 5 (large, frayed), *PC* = 6 (small, elongated, frayed), and *PC* = 7 (large, elongated, frayed). Half of the profile classes (*PC* = 0, 5, 6, 7) have favored neighborhood relations to cells of their own type. However, the preferred neighborhood relation, *PC* = 5, *NPC* = 7, which is noticeable for NScHL and MCcHL, is absent in LA.

### Small, round cells have large distances to their preferred neighbor

In NScHL, the cells have a smallest mean distance of 37.9 ± 6.1 *μm*, see Table 1. It increases to 42.7 ± 11.9 *μm* and 48.3 ± 18.1 *μm* for MCcHL and LA, respectively. Within the large standard deviations, the differences in the mean distances are not statistically significant.

**Table 1:** Mean distances of PC to the nearest neighbor in images of all diagnoses.

Diagnosis	Mean distance [*μm*]
**NScHL**	37.9 ± 6.1
**MCchl**	42.7 ± 11.9
**LA**	48.3 ± 18.1

Compared to the theoretically expected distribution for randomly located cells, the distribution of distances to the nearest neighbor was shifted towards small values and had a much more narrow shape, see S3, S4 and S5 Figs. This observation was valid for all 35 images and indicates a significant clustering of the CD30–positive cells independent of the medical diagnosis. The result has been verified by a graph-theoretical analysis of network properties of the corresponding cell graphs [36].

We asked whether the strong preference of small, round cells, *PC* = 0, to cluster together could be influenced by an attraction between these cells. For the majority of images, the ratio between the mean distances of small, round nearest neighbors and those of two nearest neighbors of arbitrary PC was greater than 1, see S6 Fig. Pairs of small, round cells showed large distances between them, with mean distances up to 50% enlarged, so that an attraction between these cells becomes unlikely. But, the preferred location of small, round cells in tissue regions that are not easily accessible by other cell types could be a possible explanation. The high motility combined with the small size of *PC* = 0 cells may lead to an aggressive ability to colonize healthy tissue regions with still sparse populations of CD30–positive cells.

The Pearson correlation coefficient of the mean distance between pairs of *PC* = *NPC* = 0 cells and the log-odd value of the significance level of a preference for these pairs were positive, over all images 0.4. The positive value of the correlation coefficient indicated that, the preference of a neighborhood of *PC* = *NPC* = 0 cells strongly correlated with a large distance between the cells.

**Fig 8:**
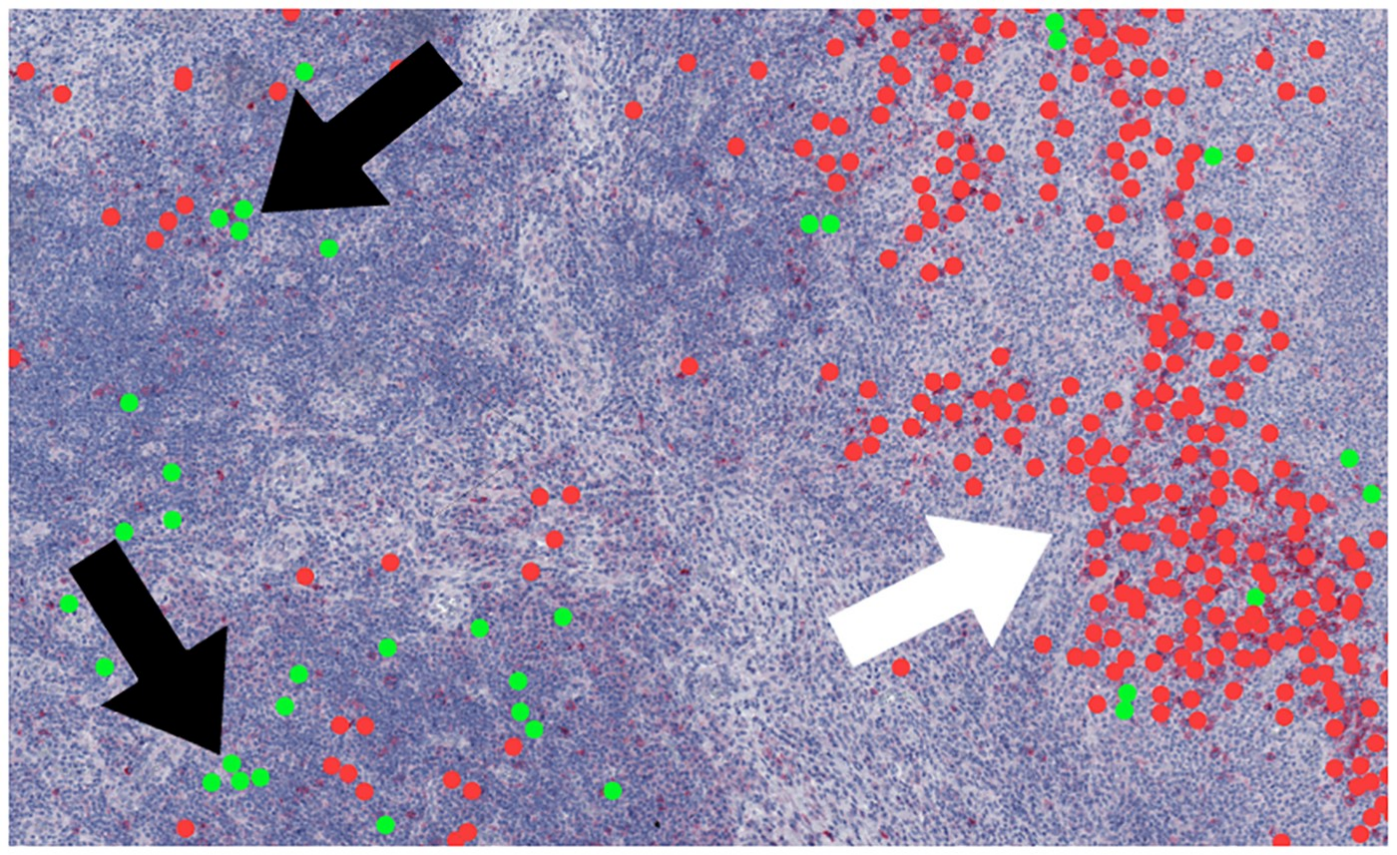
Neighborhood of small, round cells in a low density area. An exemplary subsection of an histological image with diagnosis MCcHL (ID 5722). The green points depict small, round cells (*PC* = 0) with a nearest neighbor of the same PC. The red points indicate all remaining PC. The clusters of small, round cells seem to be more often located in areas of low cell density (black arrows), whereas cell clusters of other PC seem to be more often in areas of higher cell density (white arrows).

We found a significantly high number of pairs of *PC* = *NPC* = 0 cells in tissue areas of low cell density, e.g. see Fig 8. A preferential location of small, round cells in sparsely populated tissue regions could explain the large distance between pairs of *PC* = *NPC* = 0 cells. Interestingly, a positive correlation between large distances and neighborhood preferences occurred only for these pairs, whereas for all other combinations, the correlations were negative. Only in the cases of pairs of *PC* = *NPC* = 0 cells, the preferences of cells of a PC to stay among themselves were strongly correlated with tendency to keep the distances large to the next neighbors.

## Materials and methods

### Images

We analyzed 35 two-dimensional histological WSI of human lymph nodes provided by the Dr. Senckenberg Institute of Pathology, Frankfurt am Main. The anonymized cases were manually preselected based on quality from a set of about 160 images. Because of many artifacts regarding, for example, the staining or folded tissue, the selection had to be done manually. To illustrate examples for artifacts in an image of good quality, see S7 Fig. The two depicted tissue sections have only small patches of nonspecific staining. In extreme cases, the intensity of the nonspecific staining is very close to the actively stained regions, and a large fraction of the tissue section is covered by nonspecific staining.

We chose a similar number of images for the three disease types. Overall, more MCcHL cases than lymphadenitis and NScHL cases were available. Some lymphadenitis cases show an unusual progression, e.g. an inflammation of the lymph node over a long time period. As the clinical examination is not sufficient to make the final diagnosis, the lymph node is biopsied for further histopathological exploration. Then, physicians contacted the Reference- and Consulting Center of Lymph Node and Lymphoma Pathology in Frankfurt am Main.

Additionally, the intensity of the staining was not consistently good in all areas of the image, such that even a low amount of nonspecific staining reduces the quality of the WSI significantly. The selected images cover the three histological diagnoses, NScHL (12 images), MCcHL (12 images), and LA (11 images). The tissue was CD30-immunohistochemically stained to visualize the HRS cells and special forms of activated B and T lymphocytes [37]. Overall, we considered more than 400, 000 CD30-positive cells in images with a resolution of 0.25 *μ*m × 0.25 *μ*m per pixel.

### Image processing

The images were doubly stained, one staining to visualize the cell nuclei and cytoplasm and one to label tumor cells. In immunohistologically stained images, the dye is attached to an antibody, binding to the target protein. For visualization of the cell nuclei, we used the H&E (hematoxylin & eosin) staining. Hematoxylin is a standard stain that nonspecifically binds to all negatively charged components of cells and principally colors the nuclei of cells blue or dark-purple, along with a few other tissues. Since the backbones of the DNA and RNA are the main fraction of negatively charged biomolecules within the cell, their nuclei are stained most, but also the cytosol appears in bright blue. Hematoxylin staining increases the contrast of the image and makes the cell density visible. Eosin binds to positively charged proteins and stains the cytoplasm and some other structures including extracellular matrix, such as collagen. For a review on histological staining, we refer to [38].

For identification of HRS cells, CD30 represents a typical marker protein classified by the Cluster of Differentiation (CD) protocol. The list of CD molecules consists of more than 300 unique cell surface proteins that can be targeted by monoclonal antibodies [39]. CD30 stains tumor cells of cHL and some other malignant lymphomas, e.g. anaplastic large cell lymphoma. However, CD30 is not lineage-specific and can stain any kind of activated cells, which means that reactive bystander lymphocytes can become CD30-positive when they get strongly activated. Although, cells of other tumors, which are regularly CD30-negative can become CD30-positive upon activation. The HRS cells of both cHL subtypes are positive for CD30, whereas the abundant bystander cells are CD30-negative. HRS cells secrete soluble factors, which attract bystander cells and, thus, shape their direct microenvironment [40]. This is observed in both cHL subtypes, whereas CD30-positive activated lymphocytes in lymphadenitis do not secrete the respective cytokine and, thus, differ from the malignant HRS cells. In contrast, the differential diagnosis between cHL and lymphadenitis with activated CD30-positive lymphocytes by simple morphological examination can be challenging.

### Image digitization

For digitization of the tissue samples, we used an Aperio Scanscope device and created SVS files in the Aperio’s proprietary image format that represents an altered large-tiff format, containing multiple layers. The image sizes of our samples vary from 10,000 x 10,000 pixels up to 90,000 x 90,000 pixels, see S2 Table. The image format also includes a JPEG2000 compression, resulting in SVS files with a size between 0.5 and 4 GB for images used in this work.

### Cell detection and classification

We identified cell profiles of CD30–positive cells by applying the in-house software pipeline Impro [13, 36]. Impro implements image processing methods, such as the detection of a region of interest (ROI) and the color deconvolution to separate the stains. Additional methods, such as the thresholding for the image segmentation and the computation of morphological cell descriptors, were integrated using established imaging software and libraries like CellProfiler and the Java Advanced Imaging API (JAI). S8 Fig illustrates schematically the CD30 pipeline of Impro. CellProfiler [41, 42] is an open-source image processing software focused on cell detection and images with biological background. It is designed to perform image processing tasks on high throughput data.

**Fig 9:**
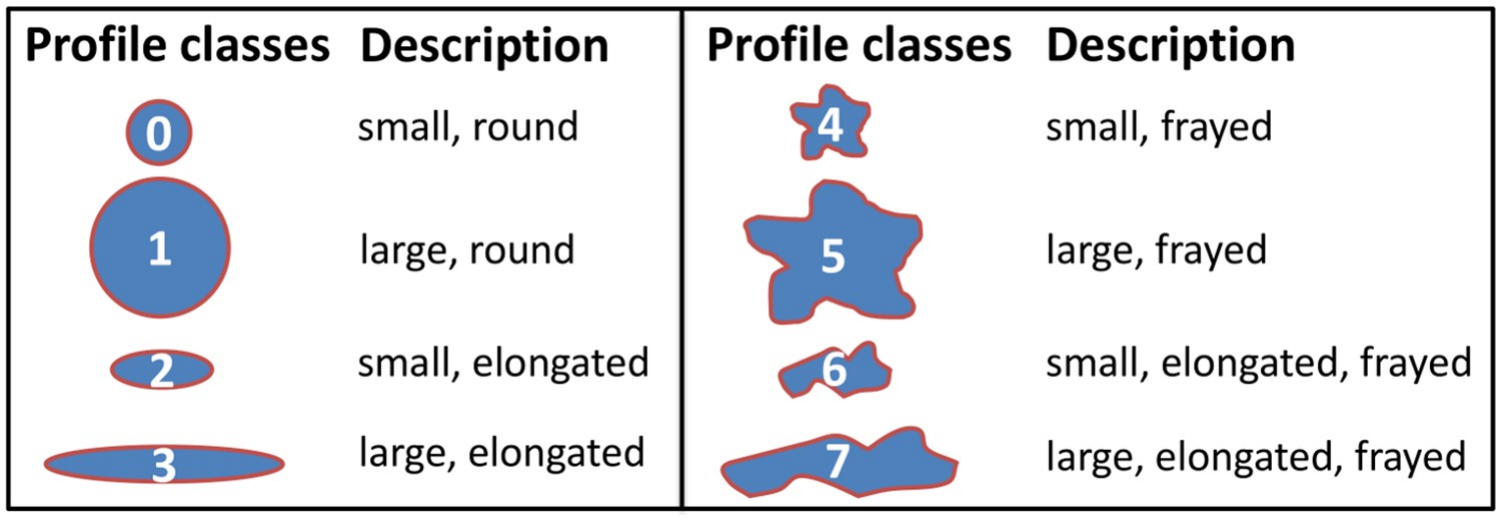
Profile classes. The eight profile classes numbered from 0 to 7 and their morphological descriptions.

The pipeline neglected small objects of size 109 *μm*^2^ or less. The threshold corresponds to the size of a round cell with a diameter of 11.8 *μm*. Applying the shape descriptors, eccentricity, solidity, and area provided by CellProfiler [41], we assigned each cell to one of the previously manually defined eight *profile classes (PC)*, see Fig 9.

The eccentricity measures the deviation of a fitted ellipse from a circle. Solidity is the fraction of the object pixels in the convex hull of the cell. Together with pathologists, we empirically defined specific thresholds to distinguish between small and large, round and elongated, and frayed and not frayed cell profiles, see S3 Table.

**Fig 10:**
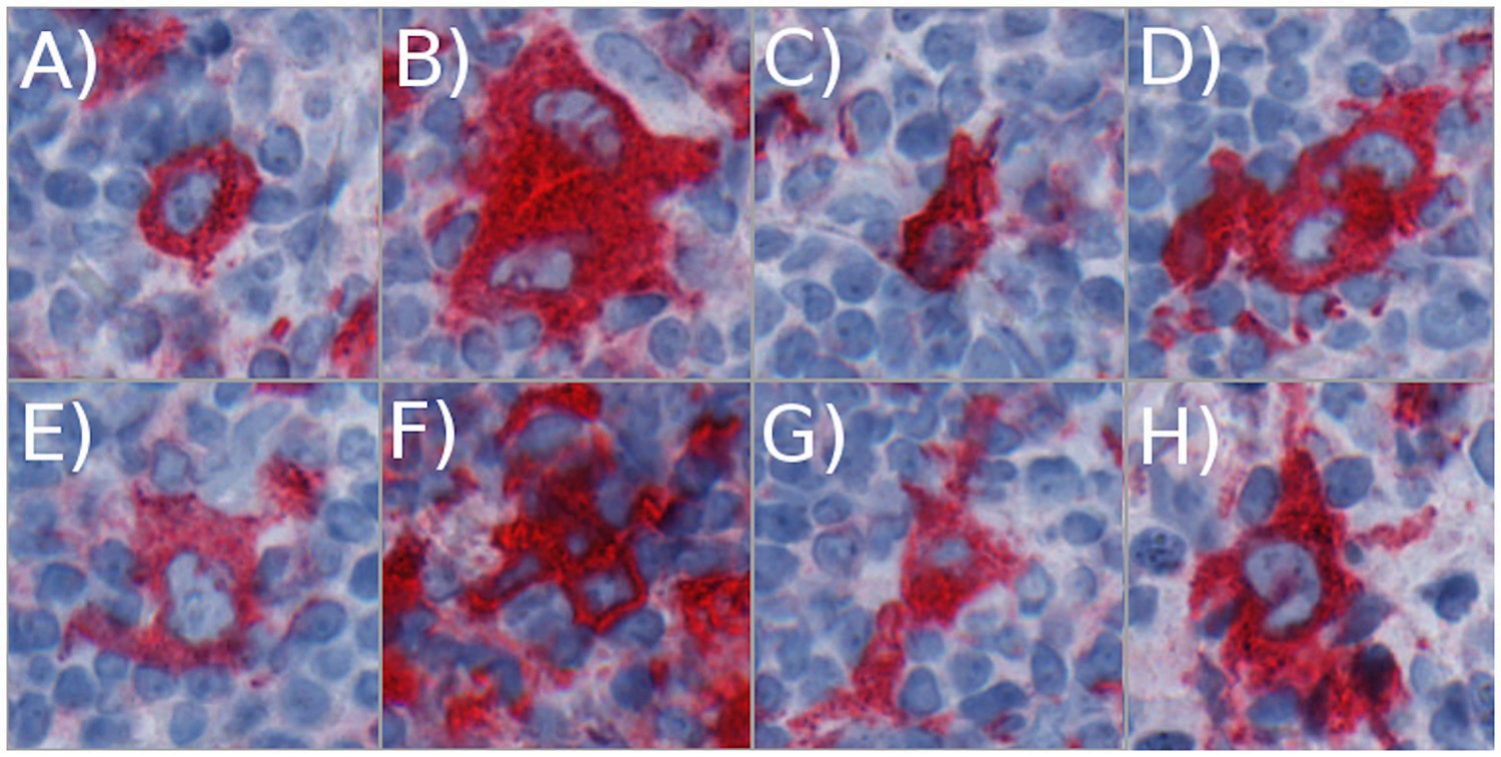
Profile classes examples. Exemplary CD30-positive cells for the eight morphologic profile classes (PC). The tissue sections display A) for PC 0 a small, round cell, B) for PC 1 a large round cell, C) for PC 2 a small, elongated cell, D) for PC 3 a large, elongated cell, E) for PC 4 a small, frayed cell, F) for PC 5 a large, frayed cell, G) for PC 6 a small, elongated, frayed cell, and H) for PC 7 a large, elongated, frayed cell.

Fig 10A) to 10H) illustrate examples for each profile class. Generally, small cell profiles are not multinucleated and, thus, could be Hodgkin cells or activated lymphocytes, whereas HRS cells are mostly multinucleated and, thus, classified and represented by big cell profiles. The size of each figure is 192 × 192 pixels with 0.25 *μm* per pixel. Thus, each figure has an edge length of 48 *μm* and covers an area of 2, 304 *μm*^2^.

For each cell profile, we computed the maximal Feret diameter using the CellProfiler module *MeasureObjectSizeShape*. The Feret diameter describes the distance of two parallel tangents to the cell in a given orientation. The maximal Feret diameter referred in the text as *diameter* is the largest value of all possible orientations.

### Neighborhood analysis

**Fig 11:**
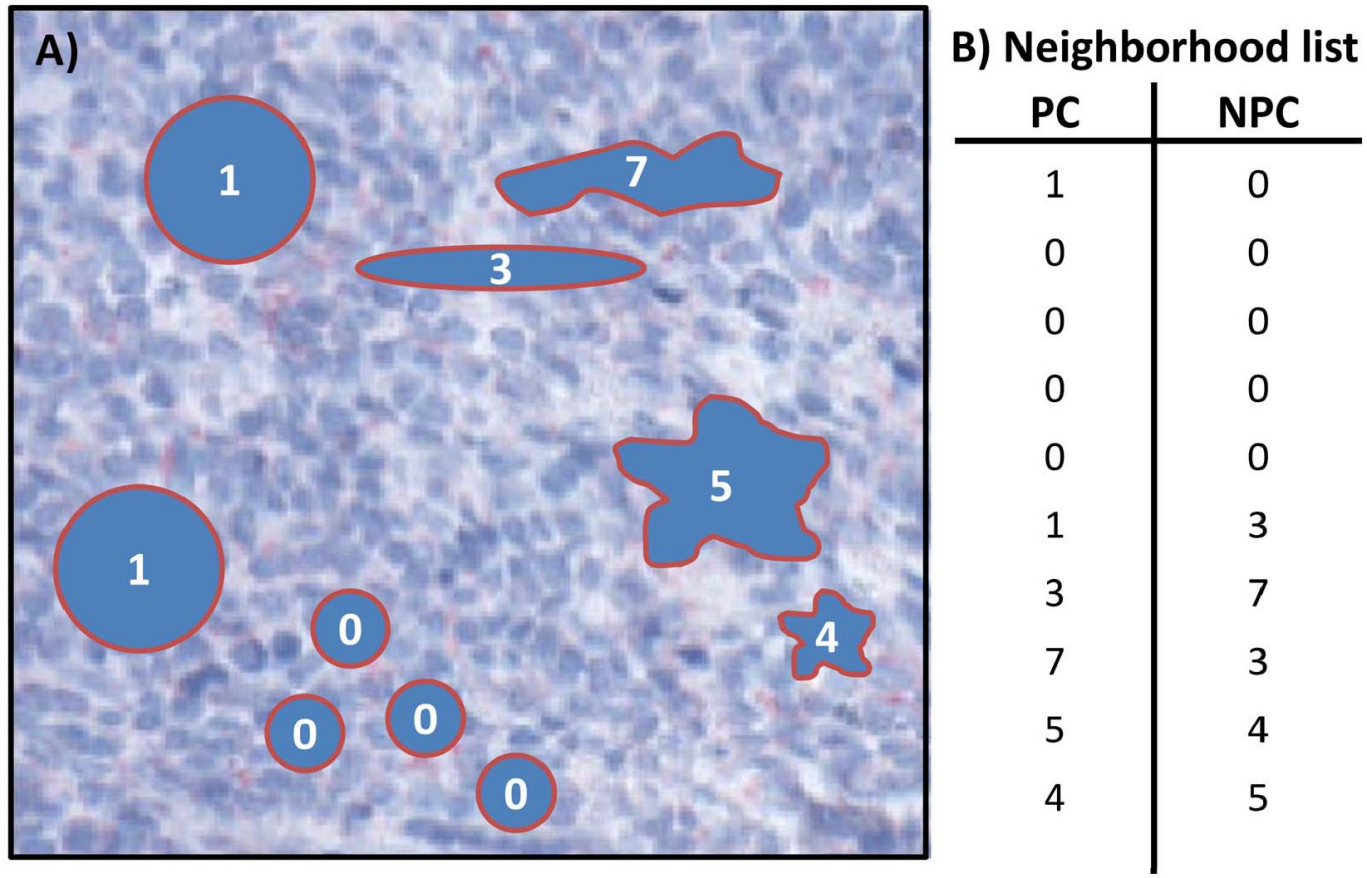
Neighborhood table. A) depicts a subsection of a schematic histological image that contains ten cells, four cells of *PC* = 0, two cells of *PC* = 1, and each single cell of *PC* = 3, 4, 5, 7, respectively. B) gives the neighborhood table that contains for each cell the PC and the PC of the nearest neighbor called *neighbor profile class (NPC)*.

To detect statistically significant correlations between cells, we studied the spatial neighborhood relationships defined by the Euclidean distance between the centers of gravity of the cells. We applied a maximal distance of 175 *μm* (700 pixels). This threshold corresponds to about ten times the diameter of an average cell, which is assumed to be sufficiently small to enable intercellular communication based on, e.g. chemokines or cytokines [43]. We computed a neighborhood table, which contains for each cell the PC and the PC of the nearest neighbor called *neighbor profile class (NPC)*, see Fig 11 for an exemplary subsection of a histological image (A) and the corresponding neighborhood table (B).

### Statistical significance

The probability to randomly choose a neighbor, *NPC* = *j*, is proportional to the frequency of the PC, *f*(*PC* = *j*), in the image. In a random choice, the PC of a cell should have no influence on the selection of the nearest neighbor. We expect to measure a value for the conditional probability that is within the statistical fluctuation indistinguishable from the relative frequency of the NPC, i.e. *P*(*NPC* = *j* | *PC* = *i*) ≈ *P*(*PC* = *j*). For example, the entry for the conditional probability, *P*(*NPC* = 0 | *PC* = 0) = 0.354, in S4(B) Table has to be compared with the entry for the expected probability, *P*(*PC* = 0) = 0.316, in S4(A) Table.

Part (A) of S4 Table exemplifies the measured probability *P*(*PC* = *i*) ≡ *f*(*i*) to find a cell of a certain PC for a given image (*ID* = 1721). The table contains the relative frequency, *f*(*i*), of the PC *i* ∈ {0, …, 7} identified by the row number.

We evaluated whether the deviation of the conditional probability from the expected probability is statistically significant for a rejection of the null hypothesis of a random selection of the nearest neighbor. For a set of *n* cells of *PC* = *i*, the probability to have a subset of *k* cells with nearest neighbor of *NPC* = *j* is given by the binomial distribution

$$P\left(k,NPC=j,PC=i\right)=B\left(k\;|\;p,n\right)=(\begin{array}{c} n\\ k \end{array})p^{k}{\left(1-p\right)}^{n-k},$$

where *p* = *P*(*PC* = *j*) is the probability to find a class, *j*, by chance, i.e. *f*(*j*), the relative frequency of class *j*. For each image and each combination of morphological classes (*PC* = *i*, *NPC* = *j*) with *i*, *j* ∈ {0, 1, …, 7}, we computed the lower and upper endpoint of the prediction interval by

$$k_{low}=max\{k\in N:\sum_{i=0}^{k}p^{i}{\left(1-p\right)}^{n-i}(\begin{array}{c} n\\ i \end{array})\leq \alpha \;/\;2\}$$

and

$$k_{up}=min\{k\in N:\sum_{i=k}^{n}p^{i}{\left(1-p\right)}^{n-i}(\begin{array}{c} n\\ i \end{array})\leq \alpha \;/\;2\}.$$

We chose a significance level of *α* = 1%. A measured value *k*, has a p-value smaller than *α* only if *k* ∉ [*k*_*low*_, *k*_*up*_]. A *k* outside the prediction interval has a significance level of *α* = 1%, which is sufficient to reject the null hypothesis of a random selection of the nearest neighbor.

For example, the image with *ID* = 1721 of diagnosis MCcHL contains 3, 435 cells of *PC* = 0 from 10, 860 cells in total. We computed the prediction interval [1047, 1125] for the significance level *α* = 1%. We measured a number of *k* = 1217 pairs of *PC* = 0 and *NPC* = 0, which is above the upper endpoint of the 1% prediction interval. Thus, we can reject the null hypothesis for the significance level *α* = 1%. Within a significance smaller than 1%, the small, round cells are enriched in the neighborhood of other small, round cells (*PC* = 0), i.e. they prefer the neighborhood of cells of their own kind. We call such a favored neighborhood relation to be *significantly high (sh)*.

If the number of measured pairs is smaller than the prediction interval for the significance level, we call the neighborhood relation to be *significantly low (sl)*. A *non-significant (ns)* neighborhood relation denotes a number of pairs within the prediction interval. We compiled the results for each of the 12 images of diagnosis NScHL, 12 images of diagnosis MCcHL, and 11 images of diagnosis LA in an individual significance matrix, e.g. see Table 2.

**Table 2:** Significance matrix for the image *ID* = 1721 of the diagnosis MCcHL.

	*PC*0	*PC*1	*PC*2	*PC*3	*PC*4	*PC*5	*PC*6	*PC*7
*NPC*0	sh					sl	sl	
*NPC*1		sh	sh	sh		sh		sh
*NPC*2			sh	sh	sl			
*NPC*3		sh		sh	sl	sh		sh
*NPC*4		sl	sl	sl	sh	sl		sl
*NPC*5		sh		sh	sl	sh		sh
*NPC*6	sl							
*NPC*7	sl	sh		sh	sl	sh		sh

PC is a profile class and NPC a neighbor profile class. The blank entries stand for ns—not significant, sh for significantly high, and sl for significantly low.

The significance matrix in Table 2 exemplifies favored and unfavored neighborhood relations for image *ID* = 1721 of diagnosis MCcHL. For example, the column for *PC* = 0 contains one entry *sh* and two entries *sl*, indicating that *PC* = 0 favors the neighborhood of cells of the same class, *NPC* = 0, but no cells of *NPC* = 6 and *NPC* = 7. The empty entries in the column for *PC* = 0 show that no favored and unfavored neighborhood relations were found between cells of *PC* = 0 and *NPC* = 1, 2, 3, 4, or 5.

For each of the 64 combinations of morphological classes, we counted the number of images, in which the combination was *sh* and *sl*, respectively. The differences of these numbers gave an integer score of neighborhood relation for each combination of classes and each diagnosis, see S2 Fig. This score of neighborhood relation gives a high positive (negative) value, when a combination of classes is *sh* (*sl*) in the majority of the images. In most cases, the neighborhood relations of cell pairs were symmetrical, e.g. see S1 Fig.

### Ethics statement

The manuscript is based on geometric cell graphs, which were computationally generated from anonymized whole slide images of human tissues and does not contain any individual person’s data in any form.

### Conclusions

With our study we demonstrated how a semi-automatic analysis of WSI of tissue can be performed. For the first time, a systematic analysis of a high number of more than 400, 000 CD30–positive cells in WSI of lymph node tissue sections of NScHL, MCcHL, and LA was performed. We provided measured values for the distances between neighbored cells, the cell diameters of HRS cells in complete lymph node sections, and morphological characteristics for each cell. The profiles of CD30–positive cells were identified by the imaging pipeline Impro [13, 36]. Based on morphological cell features, we defined eight morphological PC to classify the cells and computed the fractions of each PC. The presented study provides valuable information about the morphological characteristics and neighborhood relations of CD30-positive cells in tumor, cHL, and inflammation, LA.

Nevertheless, there are limitations of the method and many points that have to be improved. Although we considered just two disease types of Hodgkin lymphoma and one non-tumor type in 2D images and although we had not perfect material to investigate more images, we were able to perform a statistically sound analysis of CD30-positive cells. The study produced interesting results, regarding the distribution of CD30-positive cells in tumor and non-tumor tissue, the classification of these cells according to morphological properties, an analysis of the diameters of CD30-positive cells, and an analysis of favored and unfavored neighborhood relations of CD30-positive cells based on their morphological classification, all in dependence of the three considered cases, NScHL, MCcHL, and LA.

The development of different cell forms of HRS cells have been experimentally analyzed in cell cultures. Re-fusion of divided cells have been demonstrated [44]. The shape of the lymphoid cells including tumor cells is influenced by different factors. These involve the above mentioned cell proliferation, cell activation, and cell transformation, which are dynamic processes regulated and controlled by intrinsic and extrinsic factors. The phases and cell morphology reflecting cell proliferation are well known, however detailed data confining each dividing step morphologically of CD30 cells are lacking. Nondividing cells should be round whereas before cell division, a polarization indicated by cell elongation and finally resulting in two or more smaller round or irregularly shaped cells will be seen. During cell division, it seems that most cells are stationary. In G0 phase, the diameter and cell elongation as well as formation of cell processes is influenced by cell movement. During movement, cell diameters and shapes are dynamically controlled by intrinsic movement programs. Extrinsic factors of cell movement is controlled and determined by the microenvironment, as collagen fibers, sinuses, and the consistence of surfaces cells are moving on. Last but not least, the process of cell activation influences especially the cell diameter. The stronger the activation, the larger the cell looks like, as a result of pathway activations and enhanced energy consumption. Summing up, all these factors importantly configurate the appearance of the individual cells. In histological section, a snapshot including all stained cells is done freezing cell dynamic in time and space. Nevertheless, in this paper definitions of morphology and localization of CD30-positive cells is the basis for the bioinformatic computation. So we differentiated in small round, large round elongated and other cell types, including hypothetically the above described processes and principles. The results cannot be completely exact, because a dynamic model exactly reflecting these factors should be the basis of computations. This test model has to be established in the near future for further validating the results reported in this investigation. Regarding further developments, the detection of CD30–positive cells in three dimensions by applying confocal scanning microscopy [45] will be an important aspect of experimental work. Multi-staining will allow to visualize various important players beside CD30–positive cells, as e.g. T cells and B cells that are known to interact with malignant cells. Pattern recognition is and will further be a focus of ongoing research.

## Supporting information

S1 Fig A) Symmetrical neighborhood relation and B) unsymmetrical neighborhood relation. (PDF)

S2 Fig The score matrices for neighborhood relations for every combination of PC and diagnosis: A) for all 35 histological images, B) for the 12 histological images of diagnosis NScHL, C) for the 12 histological images of diagnosis MCcHL, and D) for the 11 histological images of diagnosis LA. (PDF)

S3 Fig Distribution of distances of CD30-positive cells in an image of diagnosis NScHL. (PDF)

S4 Fig Distribution of distances of CD30-positive cells in an image of diagnosis LA. (PDF)

S5 Fig Distribution of distances of CD30-positive cells in an image of diagnosis MScHL. (PDF)

S6 Fig The ratio of the mean distance of neighbors of small, round cells to the overall distance of neighbors of arbitrary type in dependence on the cell density. (PDF)

S7 Fig Artifacts in two image sections: The red arrow marks a folded tissue section often caused by inhomogeneous softness of the tissue or an unevenly worn blade. The blue arrow marks an artifact originating from enclosed air in the glass slide. Additionally, nonspecific staining may occur indicated by green arrows.
(PDF)

S8 Fig The CD30 image pipeline of the in-house Impro software. (PDF)

S1 Table Profile class mean fractions and their standard deviations for all 35 images with respect to the diagnosis. (PDF)

S2 Table Image sizes of all 35 images with respect to the diagnosis. (PDF)

S3 Table Profile class definitions. (PDF)

S4 Table Probabilities and conditional probabilities. (PDF)
