# Supplementary figures and images for: Bioinformatics analysis of whole slide images reveals significant neighborhood preferences of tumor cells in Hodgkin lymphoma
Source: PLoS Comput Biol. 2020 Jan 21;16(1):e1007516. doi: 10.1371/journal.pcbi.1007516 (PMC6999891; doi:10.1371/journal.pcbi.1007516)

## A) Symmetrical neighborhood relation

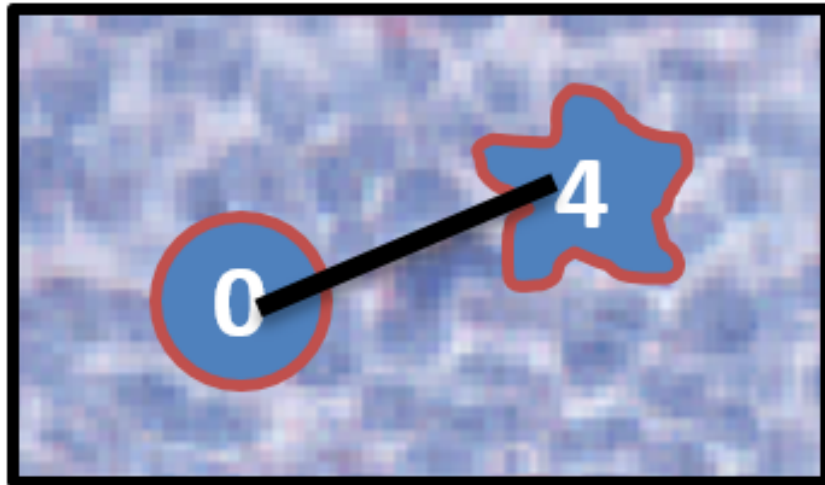

| CP | NCP |
|----|-----|
| 0  | 4   |
| 4  | 0   |

## B) Unsymmetrical neighborhood relation

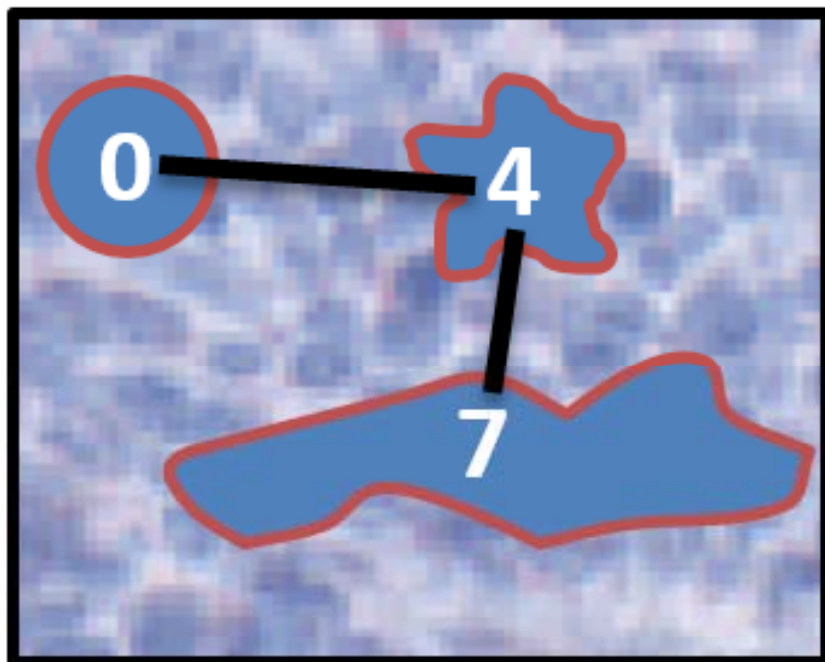

| CP | NCP |
|----|-----|
| 0  | 4   |
| 4  | 7   |
| 7  | 4   |

Supplement: S1 Fig — (PDF) [file pcbi.1007516.s001.pdf]

image 2952(NScHL)

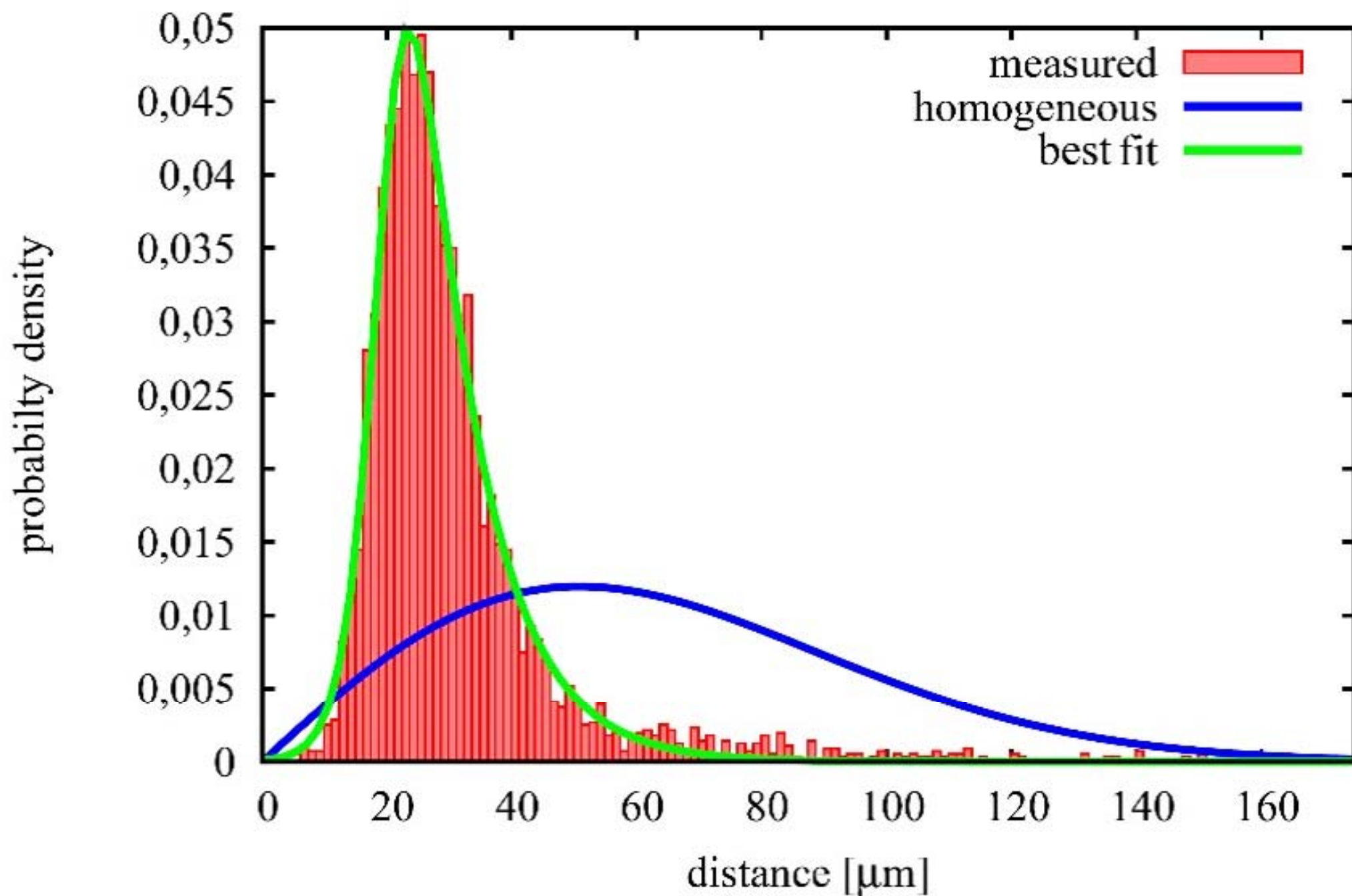

Supplement: S3 Fig — (PDF) [file pcbi.1007516.s003.pdf]

image 5561(LA)

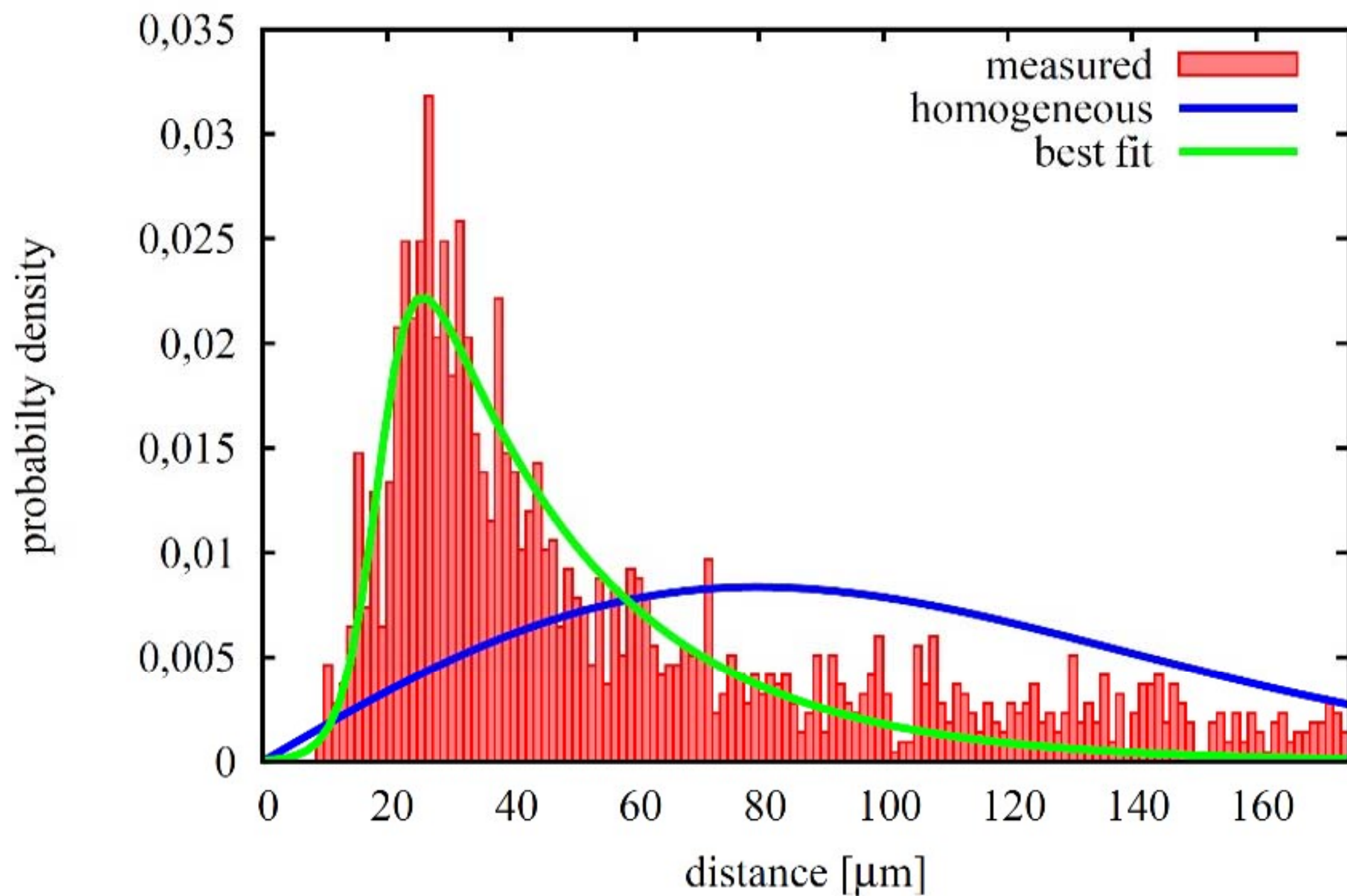

Supplement: S4 Fig — (PDF) [file pcbi.1007516.s004.pdf]

image 7486 (MCcHL)

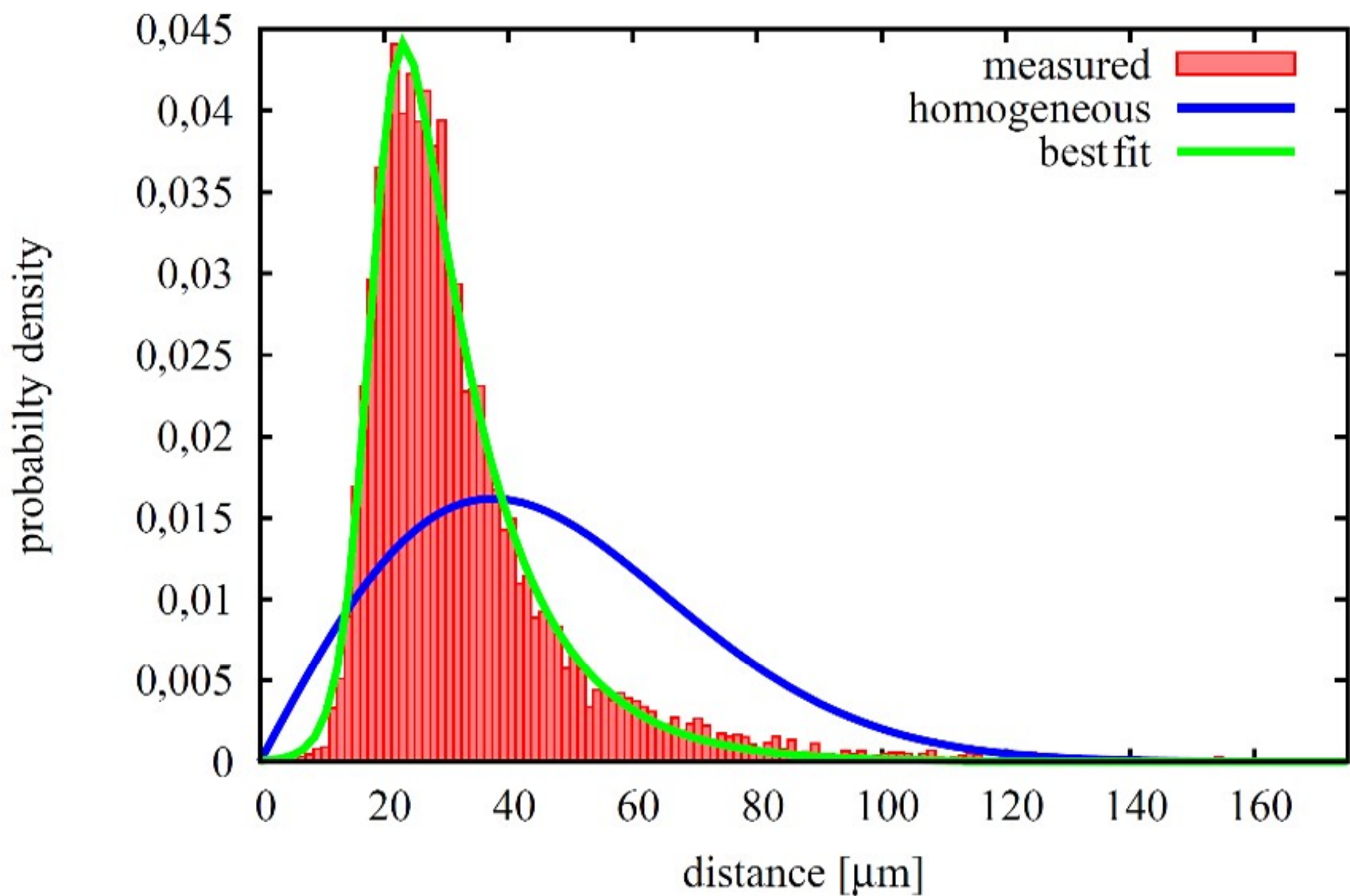

Supplement: S5 Fig — (PDF) [file pcbi.1007516.s005.pdf]

mean distance [ $\mu\text{m}$ ]

random  
LA  
MCcHL  
NScHL

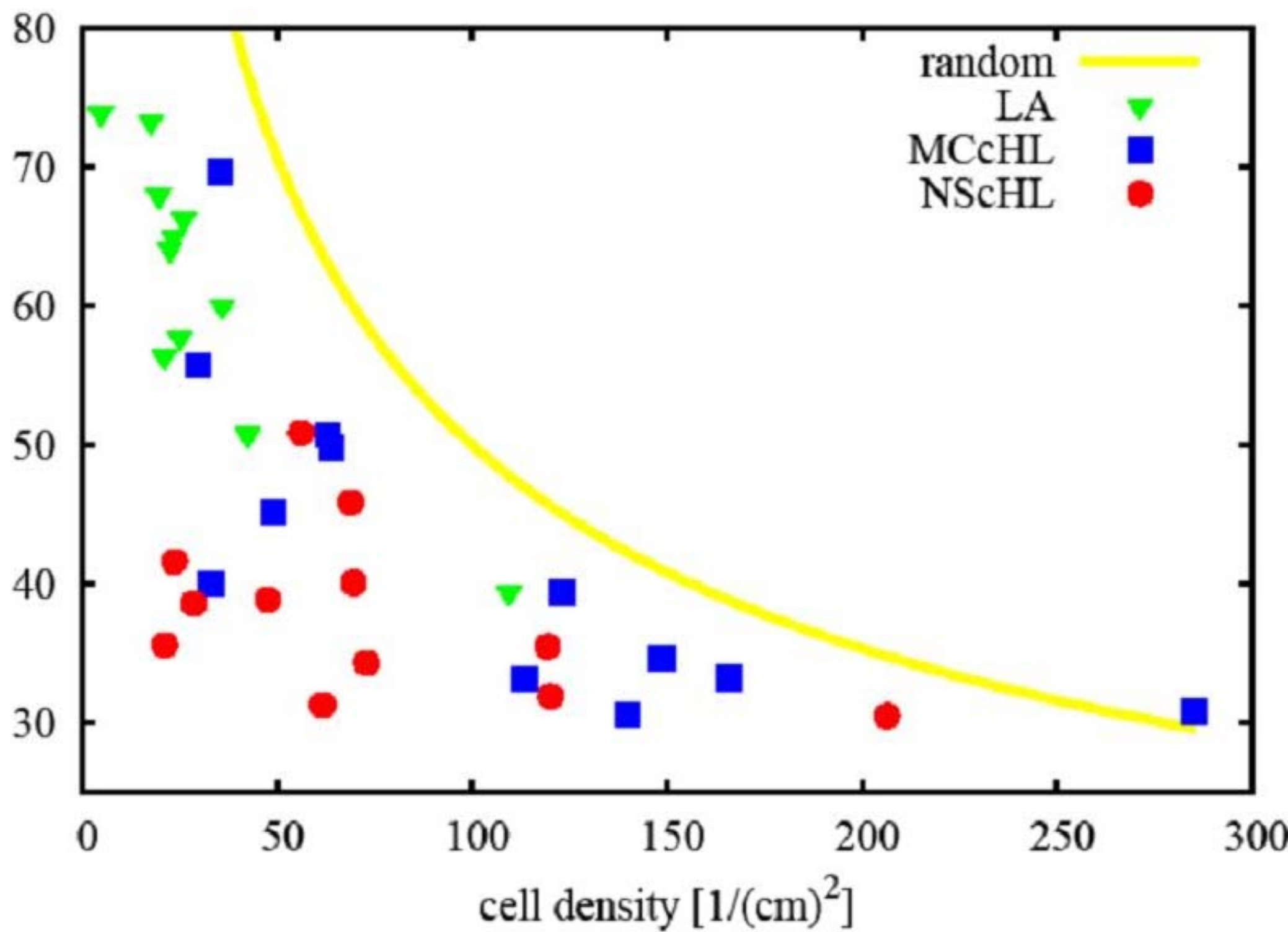

Supplement: S6 Fig — (PDF) [file pcbi.1007516.s006.pdf]

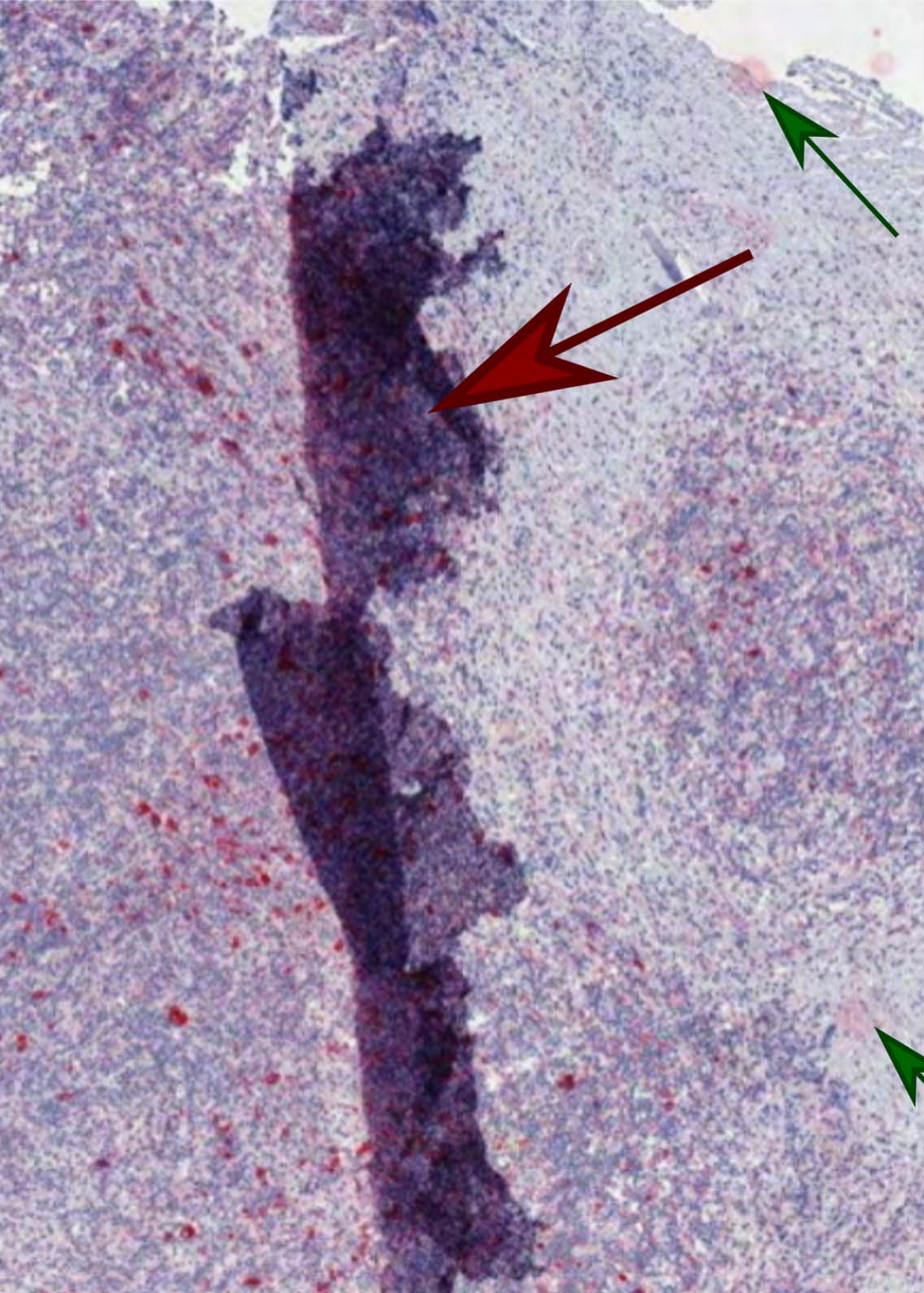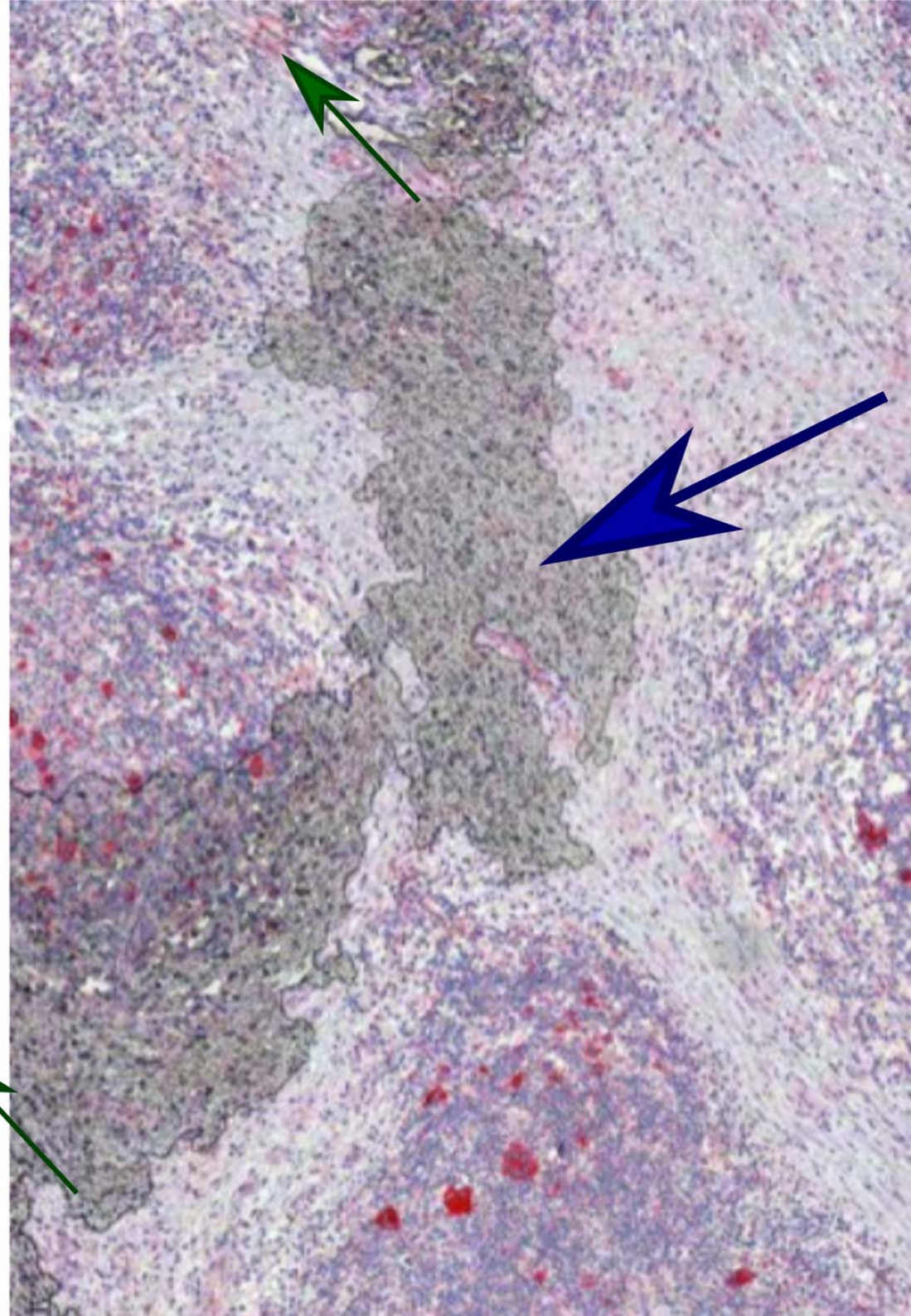

Supplement: S7 Fig — The blue arrow marks an artifact originating from enclosed air in the glass slide. Additionally, nonspecific staining may occur indicated by green arrows. (PDF) [file pcbi.1007516.s007.pdf]

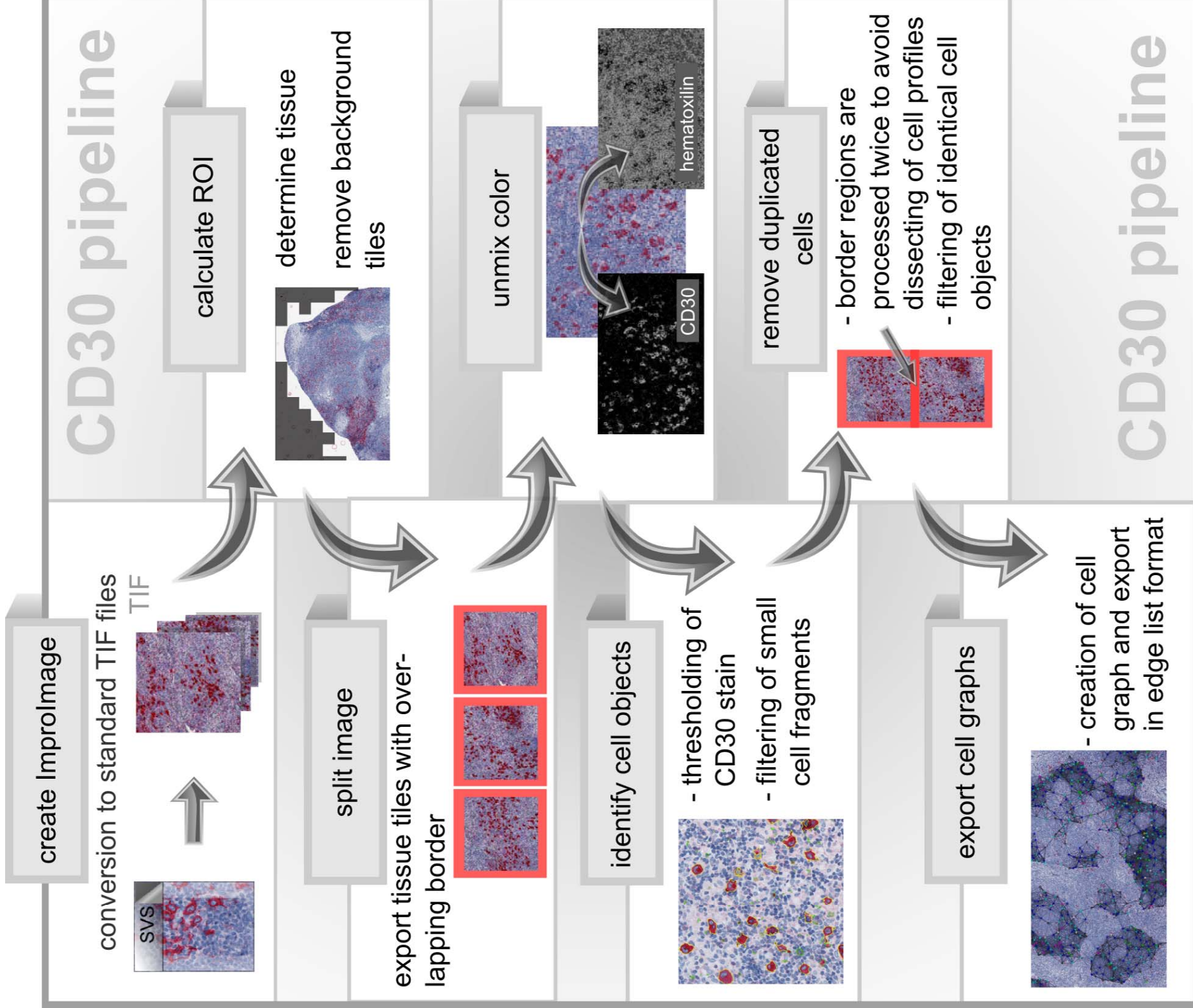

Supplement: S8 Fig — (PDF) [file pcbi.1007516.s008.pdf]
