# Supplementary material for: Bioinformatics analysis of whole slide images reveals significant neighborhood preferences of tumor cells in Hodgkin lymphoma
Source: PLoS Comput Biol. 2020 Jan 21;16(1):e1007516. doi: 10.1371/journal.pcbi.1007516 (PMC6999891; doi:10.1371/journal.pcbi.1007516)
Supplement: S2 Fig — (PDF) [file pcbi.1007516.s002.pdf]

A) 35 histological images

| | 0 | 1 | 2 | 3 | 4 | 5 | 6 | 7 |
|---|---|---|---|---|---|---|---|---|
| **0** | 31 (89%) | -6 (17%) | 4 (11%) | -3 (9%) | -15 (43%) | -18 (51%) | -26 (74%) | -18 (51%) |
| **1** | 0 | 14 (40%) | -1 (3%) | 7 (20%) | -6 (17%) | 8 (23%) | -6 (17%) | 8 (23%) |
| **2** | 5 (14%) | -1 (3%) | 7 (20%) | 1 (3%) | -3 (9%) | -5 (14%) | 0 | 1 (3%) |
| **3** | -1 (3%) | 5 (14%) | 0 | 6 (17%) | -4 (11%) | 4 (11%) | -1 (3%) | 2 (6%) |
| **4** | -9 (26%) | -8 (23%) | -4 (11%) | -6 (17%) | 17 (49%) | -1 (3%) | 9 (26%) | 5 (14%) |
| **5** | -15 (43%) | 10 (29%) | -2 (6%) | 2 (6%) | 1 (3%) | 19 (54%) | -3 (9%) | 20 (57%) |
| **6** | -24 (69%) | -8 (23%) | 2 (6%) | -3 (9%) | 9 (26%) | 0 | 23 (66%) | 3 (9%) |
| **7** | -13 (37%) | 6 (17%) | -1 (3%) | 2 (6%) | -1 (3%) | 15 (43%) | 3 (9%) | 15 (43%) |

B) 12 histological images diagnosed as NScHL

| | 0 | 1 | 2 | 3 | 4 | 5 | 6 | 7 |
|---|---|---|---|---|---|---|---|---|
| **0** | 10 (83%) | -1 (8%) | 0 | -1 (8%) | -5 (42%) | -4 (33%) | -8 (67%) | -5 (42%) |
| **1** | 1 (8%) | 7 (58%) | 0 | 3 (25%) | -3 (25%) | 3 (25%) | -2 (17%) | 3 (25%) |
| **2** | 1 (8%) | -1 (8%) | 2 (17%) | 0 | 0 | -2 (17%) | 0 | -1 (8%) |
| **3** | 1 (8%) | 1 (8%) | 0 | 3 (25%) | -2 (17%) | 1 (8%) | 0 | 1 (8%) |
| **4** | -2 (17%) | -3 (25%) | -1 (8%) | -3 (25%) | 6 (50%) | -1 (8%) | 2 (17%) | -3 (25%) |
| **5** | -4 (33%) | 3 (25%) | 0 | 0 | 1 (8%) | 6 (50%) | -1 (8%) | 8 (67%) |
| **6** | -7 (58%) | -4 (33%) | 1 (8%) | -2 (17%) | 4 (33%) | 0 | 7 (58%) | 1 (8%) |
| **7** | -2 (17%) | 2 (17%) | 0 | 1 (8%) | -1 (8%) | 7 (58%) | 1 (8%) | 2 (8%) |

C) 12 histological images diagnosed as MCcHL

| | 0 | 1 | 2 | 3 | 4 | 5 | 6 | 7 |
|---|---|---|---|---|---|---|---|---|
| **0** | 11 (92%) | 1 (8%) | 3 (25%) | 1 (8%) | -6 (50%) | -6 (50%) | -8 (67%) | -5 (42%) |
| **1** | 1 (8%) | 3 (25%) | 0 | 2 (17%) | -2 (17%) | 1 (8%) | -3 (25%) | 2 (17%) |
| **2** | 2 (17%) | 0 | 4 (33%) | 1 (8%) | -3 (25%) | -2 (17%) | 0 | 0 |
| **3** | 0 | 2 (17%) | 0 | 2 (17%) | -2 (17%) | 1 (8%) | -1 (8%) | 1 (8%) |
| **4** | -3 (25%) | -3 (25%) | -3 (25%) | -3 (25%) | 7 (58%) | -1 (8%) | 4 (33%) | -2 (17%) |
| **5** | -5 (42%) | 3 (25%) | -2 (17%) | 1 (8%) | -1 (8%) | 6 (50%) | -1 (8%) | 7 (58%) |
| **6** | -7 (58%) | -3 (25%) | 1 (8%) | -2 (17%) | 4 (33%) | 0 | 8 (67%) | -1 (8%) |
| **7** | -4 (33%) | 2 (17%) | -1 (8%) | 1 (8%) | -1 (8%) | 3 (25%) | -1 (8%) | 5 (42%) |

D) 11 histological images diagnosed as LA

| | 0 | 1 | 2 | 3 | 4 | 5 | 6 | 7 |
|---|---|---|---|---|---|---|---|---|
| **0** | 10 (91%) | -6 (55%) | 1 (9%) | -3 (27%) | -4 (36%) | -8 (73%) | -10 (91%) | -8 (73%) |
| **1** | -2 (18%) | 4 (36%) | -1 (9%) | 2 (18%) | -1 (9%) | 4 (36%) | -1 (9%) | 3 (27%) |
| **2** | 2 (18%) | 0 | 1 (9%) | 0 | 0 | -1 (9%) | 0 | 0 |
| **3** | -2 (18%) | 2 (18%) | 0 | 1 (9%) | 0 | 2 (18%) | 0 | 0 |
| **4** | -4 (36%) | -2 (18%) | 0 | 0 | 4 (36%) | 1 (9%) | 3 (27%) | 0 |
| **5** | -6 (55%) | 4 (36%) | 0 | 1 (9%) | 1 (9%) | 7 (64%) | -1 (9%) | 5 (45%) |
| **6** | -10 (91%) | -1 (9%) | 0 | 1 (9%) | 1 (9%) | 0 | 8 (73%) | 3 (27%) |
| **7** | -7 (64%) | 2 (18%) | 0 | 0 | 1 (9%) | 5 (45%) | 3 (27%) | 7 (64%) |
