# Supplementary material for: Bioinformatics analysis of whole slide images reveals significant neighborhood preferences of tumor cells in Hodgkin lymphoma
Source: PLoS Comput Biol. 2020 Jan 21;16(1):e1007516. doi: 10.1371/journal.pcbi.1007516 (PMC6999891; doi:10.1371/journal.pcbi.1007516)
Supplement: S1 Table — (PDF) [file pcbi.1007516.s009.pdf]

Profile class (PC) mean fractions and their standard deviations for all 35 images and with respect to the diagnosis.

PC	All images [%]	NScHL [%]	MCcHL [%]	LA [%]
0	38.63 ±16.03	30.93 ±10.51	38.32 ±18.30	47.36 ±15.13
1	2.63 ± 2.86	3.72 ± 2.55	1.79 ± 1.39	2.37 ± 4.03
2	5.23 ± 2.03	5.16 ± 2.09	5.88 ± 2.36	4.58 ± 1.43
3	0.75 ± 0.87	1.13 ± 0.93	0.55 ± 0.41	0.57 ± 1.08
4	25.47 ± 8.66	28.22 ± 8.59	27.38 ± 9.3	20.39 ± 5.92
5	4.98 ± 4.32	7.39 ± 3.73	5.09 ± 5.21	2.21 ± 1.71
6	18.38 ± 7.14	18.32 ± 6.28	17.36 ± 5.96	19.56 ± 9.37
7	3.93 ± 3.17	5.12± 2.53	3.63 ± 3.32	2.95 ± 3.50
