# Supplementary material for: Bioinformatics analysis of whole slide images reveals significant neighborhood preferences of tumor cells in Hodgkin lymphoma
Source: PLoS Comput Biol. 2020 Jan 21;16(1):e1007516. doi: 10.1371/journal.pcbi.1007516 (PMC6999891; doi:10.1371/journal.pcbi.1007516)
Supplement: S2 Table — (PDF) [file pcbi.1007516.s010.pdf]

The image sizes of all 35 images with respect to the diagnosis

NScHL		MCcHL		LA	
SVS id	sqrt(roi) [px]	SVS id	sqrt(roi) [px]	SVS id	sqrt(roi) [px]
7489	48378	5617	71885	6275	40857
7228	45977	7082	62051	6277	35825
6292	38205	5800	24405	5558	36636
6286	30515	6643	52773	5566	38315
7480	52853	5824	58993	5559	47942
7358	51892	5722	41266	5565	44588
5070	64626	7486	47844	5556	60407
2952	34392	5822	55097	5561	34996
2961	66489	5796	66228	5557	40028
2140	53327	6267	56534	5161	36175
6637	56087	1721	53080	5563	65855
1724	63785	2949	32896		
