# Supplementary material for: Bioinformatics analysis of whole slide images reveals significant neighborhood preferences of tumor cells in Hodgkin lymphoma
Source: PLoS Comput Biol. 2020 Jan 21;16(1):e1007516. doi: 10.1371/journal.pcbi.1007516 (PMC6999891; doi:10.1371/journal.pcbi.1007516)
Supplement: S3 Table — (PDF) [file pcbi.1007516.s011.pdf]

The profile classes (PC) of cells, small and large, round and elongated, and frayed and not frayed. We empirically defined these classes with pathologists, using the measures eccentricity, solidity, and area of an object as provided by CellProfiler. We consider as object an immunohistochemically stained cell. For eccentricity, we consider a fitted ellipse that covers the cell. Then, the eccentricity of the ellipse is the ratio of the distance of two foci divided by the length of the major axis of the ellipse. Solidity is the ratio of the cell area and the convex hull of the cell. Area is the area of the cell based on pixel assignment. We directly converted the pixel number with 0.25 μm x 0.25 μm according to the resolution of a pixel.

PC	Description	Eccentricity	Solidity	Area [μm^2]
0	small, round	< 0.85	> 0.75	< 500
1	large, round	< 0.85	> 0.75	> 500
2	small, elongated	> 0.85	> 0.75	< 500
3	large, elongated	> 0.85	> 0.75	> 500
4	small, frayed	< 0.85	< 0.75	< 500
5	large, frayed	< 0.85	< 0.75	> 500
6	small, elongated, frayed	> 0.85	< 0.75	< 500
7	large, elongated, frayed	> 0.85	< 0.75	> 500
