# Supplementary material for: Bioinformatics analysis of whole slide images reveals significant neighborhood preferences of tumor cells in Hodgkin lymphoma
Source: PLoS Comput Biol. 2020 Jan 21;16(1):e1007516. doi: 10.1371/journal.pcbi.1007516 (PMC6999891; doi:10.1371/journal.pcbi.1007516)
Supplement: S4 Table — (PDF) [file pcbi.1007516.s012.pdf]

Probabilities and conditional probabilities. PC stands for profile class and NPC for neighbor profile class. Part (A): The eight entries give the relative frequency of each PC in an image of the diagnosis MCcHL (ID = 1721). For example, $31,6\%$ of the $10,860$ cells are small, round (PC = 0). Part (B): The 64 entries give the conditional probabilities, $P(\text{NPC} = j \mid \text{PC} = i)$, to find an NPC $= j$ for a PC $= i$. The conditional probability is approximated by $P(\text{NPC} = j \mid \text{PC} = i) = f(i,j)/f(i)$, where $f(i,j)$ is the frequency of pairs $(PC = i, NPC = j)$ in the neighborhood list. For example, the nearest neighbor of a small, round PC (PC = 0) is as well small, round (NPC = 0) with a probability of 0.354.

(A)

PC	0	1	2	3	4	5	6	7
	.316	.017	.046	.012	.376	.015	.199	.018

(B)

PC	0	1	2	3	4	5	6	7
NPC								
0	.354	.246	.323	.248	.308	.226	.289	.202
1	.013	.110	.016	.105	.012	.067	.015	.071
2	.049	.033	.077	.113	.036	.067	.053	.081
3	.009	.076	.026	.068	.007	.055	.015	.066
4	.384	.262	.289	.195	.418	.213	.374	.242
5	.011	.055	.020	.053	.007	.128	.009	.045
6	.167	.126	.228	.135	.203	.158	.221	.157
7	.012	.093	.022	.083	.009	.085	.020	.136
